# Impact of the COVID-19 pandemic on university performance: a retrospective follow-up study of the University of Split, Croatia

**DOI:** 10.7189/jogh.16.04017

**Published:** 2026-01-12

**Authors:** Jelena Hrga, Antonija Mijatović, Dragan Ljutić, Ana Marušić

**Affiliations:** 1 Rectorate, University of Split, Split, Croatia; 2 Department of Research in Biomedicine and Health, University of Split School of Medicine, Split, Croatia

## Abstract

**Background:**

The COVID-19 pandemic has affected the academic performance, financial health, scientific output, and student and staff mobility of higher education institutions globally. Here, we report on a retrospective analysis of the core activities at the University of Split, Croatia, from 2017 to 2023, with a focus on the pandemic’s impact thereon.

**Methods:**

Using interrupted time series analysis, we examined trends in student success, research output, financial indicators, and mobility patterns before, during, and after the pandemic, with a total of 34 indicators.

**Results:**

We found no significant disruptions in academic performance, financial stability, or overall institutional operations at the university level, while the observed differences at the faculty level were unrelated to the COVID-19 pandemic.

**Conclusions:**

These findings indicate that, in the observed period, the University of Split did not experience measurable pandemic-related disruptions in key academic, financial, and operational indicators. They emphasise the importance of institutional preparedness, digital adaptability, and financial diversification to ensure the stability and resilience of higher education institutions in future crises.

The COVID-19 outbreak, proclaimed a pandemic by the World Health Organization in March 2020 [1], caused extensive disruptions to higher education institutions (HEIs) throughout the world. The ensuing transition to distance learning and remote work created major difficulties for students and staff members, as well as institutional management [2,3]. Members of university communities, including students and academic staff, experienced higher stress levels of anxiety and depression, together with decreased student motivation and intensified social inequalities throughout this period [4,5]. Financially, HEIs suffered losses as the pandemic cut off their revenue streams, forcing them to implement new support systems for students and staff [6]. While several studies have investigated the impact of the pandemic on students and staff [7,8], systematic institutional-level analyses remain limited.

In response to the COVID-19 pandemic, universities worldwide have faced significant challenges and have implemented various strategies to mitigate the impact on students, faculty, and staff [9,10]. Problems with technology and poor quality of courses negatively affected students’ motivation [11–14]. The number of students enrolling in universities has also been affected, with some students opting to defer their studies due to the uncertainties brought about by the pandemic [15,16]. The pandemic also exacerbated existing social inequalities among students. At German higher education institutions, for example, Kopmann and colleagues [17] found that particularly vulnerable student groups, such as students with disabilities and students with children, were more likely to drop out their colleagues. Responses at the university level, meanwhile, included the establishment of support teams to navigate COVID-19 policies and procedures [18], the implementation of online learning platforms, and the development of crisis response strategies to sustain universities’ reputations, as perceived by the general public [19].

The pandemic affected researchers’ productivity in heterogeneous ways across disciplines and demographic groups. Several studies reported gender disparities, with female researchers experiencing greater challenges related to increased caregiving responsibilities, school closures, and the shift to remote work [20,21]. Restrictions such as school closures and social distancing measures have disproportionately affected female researchers due to limitations in childcare options and the shift to remote work [22]. Addressing such disparities has been highlighted as important for supporting equity in academic research, particularly during crises [23,24]. Simultaneously, publication output increased in certain fields and institutional contexts. For example, a rise in newly published articles by top US departments was reported in economics and finance journals [25], while increased scientific publication output was also observed in biomedicine [26].

Universities reliant on international student fees and partnerships were likewise affected by the pandemic [6]. Several higher education systems saw significant revenue losses, particularly universities reliant on international student fees, resulting in the implementation of measures such as salary reductions [27]. In response, many institutions implemented student support programmes, including food vouchers, transportation subsidies, and online learning equipment, some of which were funded by emergency government assistance [28].

The pandemic also reversed the previously rising trends of student mobility and international university collaborations, stopping exchange programmes, internships, and travel due to government restrictions aimed at controlling the spread of the virus [29–31]. It also accelerated the adoption of virtual mobility initiatives as part of a broader digital transformation in higher education, supporting hybrid educational models and the expansion of digital infrastructure [32–35]. At the same time, disruptions to physical mobility were accompanied by changes in students’ interest in studying abroad. A study conducted in 2021 in China and Hong Kong showed that 84% of students showed no interest in studying abroad after the pandemic or changed their planned destination [36].

The literature on resilience in higher education mainly centres on digital infrastructure and institutions’ ability to shift to online learning environments [37]. During the pandemic, many universities quickly changed their assessment strategies and teaching methods, with studies suggesting that this emergency shift could lead to lasting innovations in pedagogy and evaluation practices [38]. Many institutions established *ad hoc* crisis management or coordination teams to ensure academic continuity, although institutional capacity significantly influenced the effectiveness of these responses [39].

Our aim was to assess the impact of the COVID-19 pandemic on the financial and academic performance of the University of Split. Specifically, through a retrospective study, we wanted to compare the University’s enrolment patterns, student achievement rates, research output, academic exchange and financial performance during the COVID-19 pandemic compared to the pre-pandemic period, *i.e.* from 2017 to 2023.

## METHODS

### Setting

The University of Split, the second largest institution of its kind in Croatia, is a medium-sized, publicly funded European university that serves about 20 000 undergraduate, graduate, and postgraduate students. It consists of 11 faculties, an Academy, and the Rectorate as a central unit. Except for four departments integrated within the Rectorate, each faculty and the academy, despite being subsumed within the University, act as separate legal entities. For this reason, the faculties and the academy develop standalone reports; the four departments, while not obligated to draft official financial reports, are monitored by the Rectorate´s business books.

In line with the recommendation of the Croatian Institute of Public Health, the Rector of the University decided to suspend regular in-person teaching activities on 16 March 2020 at the start of the COVID-19 pandemic, and implemented a new teaching regime (distance learning model).

### Data sources

We used four official datasets stored in the University of Split’s repositories, combining them according to two different temporal structures: academic years (used for student, mobility, and educational indicators) and fiscal or calendar years (used for financial and research output indicators). There were two reasons for this analytical decision. First, it ensured consistency in pre- and post-pandemic comparisons within each indicator category, as academic and fiscal year reporting cycles are fixed, institutionally defined, and stable over time. Second, we sought not to synchronise all indicators into a single time base, but to assess whether each domain shows evidence of disruption around the onset of COVID-19 within its own reporting structure. Therefore, this temporal misalignment between indicators did not bias trend detection, as all comparisons were internally consistent within each dataset.

#### Academic indicators for each academic year

This dataset was extracted from the Student’s Success Analysis, an official report drafted for every academic year by the Quality Department of the Rectorate, which includes the indicators of students' performance:

– the total number of students enrolled per academic year from 2016/2017 to 2022/2023;

– the number of students who graduated at bachelor's and master's degrees levels;

– the number of students who fulfilled requirements for higher study year (>60 European Credit Transfer and Accumulation System (ECTS) points);

– the number of students who did not fulfil requirements for a higher study year (<42 ECTS points);

– the number of students who gave up studies in first study year; the number of students who gave up studies at any time.

The ECTS points, which we used to measure students' success, are a standardised credit system adopted by 49 countries participating in the Bologna Process. Croatia implemented this process in 2005, aligning all higher education programmes with the European Higher Education Area. Students who achieved more than 60 ECTS points in an academic year fulfil requirements for a higher study year and are considered more successful than the others, while those who achieved less than 42 ECTS points do not.

We also retrieved data on students subject to public subsidies (in EUR) from the Rectorate’s Office for Education. As the University is a publicly funded organisation, it receives public subsidies for students enrolled in the study programme for the first time and for students who achieved more than 55 ECT points in their senior years (or more than 30 ECT points if they have disabilities). The amounts of public subsidies differ across scientific fields and are defined through Governmental decisions for each academic year. The amount of public subsidy refers to the academic year, not the fiscal business year, so we decided to display these values as a part of the academic indicators.

#### Academic staff and scientific output for calendar years

Scientific productivity/output was measured based on the number of journal articles published by authors affiliated with the University of Split between 2017 and 2022 that were indexed in the Web of Science Core Collection and Scopus. Since many publications include authors from multiple faculties, with multiple affiliations to the University, we used fractional counting to prevent artificially inflating publication totals in units with higher collaboration levels. Each publication was divided proportionally among the contributing faculties, resulting in decimal values, and thus providing a more precise view of faculty-level output. Papers were categorised into the following disciplines: science, technology, engineering, and mathematics + biomedicine and health care (STEM + health), social sciences and humanities (SSH), and the arts (ART). Alongside the number of published scientific papers, we tracked the number of employees holding a scientific-teaching position per each scientific discipline.

All these data were obtained from the Rectorate’s Science Office, which collects, analyses, and consolidates data on research and coordinates scientific activities at the University level.

#### Financial indicators for calendar (business) years

We retrieved the main income and expenditure items from the official financial reports submitted to the Ministry of Finance on an annual basis. Business revenues within the dataset were generated from normal business operations of the University and included payments received from students or any other subject, *i.e.* public sector, or physical or legal person.

The revenues from tuition fees (*i.e.* payments of students who are not eligible for state subsidy) and from market operations (*i.e.* from providing services on the market) were separately highlighted and disclosed within the dataset. Students who had achieved 55 ECTS points in the previous academic year do not pay any fees; those achieving less than 30 ECTS points pay the full tuition fee; those achieving between 30 and 55 ECTS points pay the difference up to the number of ECTS enrolled in that academic year.

Revenues from public subsidies are also part of business revenues, but they were presented in the Section One data set, together with data on academic indicators, because those subsidies are associated with student success and refer to academic, not fiscal business years. Revenues from the sale of non-financial assets are collected from the sale of property/assets (real estate, equipment, *etc*.). Revenues from financial assets and borrowing are generated from loans given to non-profit organisations and legal or physical persons. Business expenditures represent all expenses related to normal business operations (salaries, material costs, *etc*.). Expenditures for business trips were separately disclosed, as they were especially sensitive to the circumstances of the pandemic. Expenditure for the purchase of non-financial assets includes funds outflow for the purchase of long-term assets (real estate, equipment, *etc*.). Expenditures for loan repayments represent outflow of funds due to loan servicing. Business results as an indicator of business success at the end of the business year and can be disclosed as surplus or deficit of revenues. Average number of employees is given in financial reports as the average number of employees at the beginning and end of the reporting period.

We also measured two other financial indicators. Specifically, the proportion of public subsidies in total revenue serves as a key indicator in the public higher education finance sector. It how much an institution depends on government financial streams, which are usually the most stable source of income for public universities. We calculated the annual percentages as the ratio of public subsidies to total institutional revenue. The annual surplus or deficit, measured as a percentage of total revenue, reflects the institution’s operating balance after all expenses, expressed relative to its total income, and is commonly used to evaluate fiscal sustainability in publicly funded higher education systems. Stable or slightly positive ratios suggest consistent budget management and resilience to external shocks [40].

##### Incoming and outgoing mobilities of staff and students for academic year

This dataset, managed by the Rectorate’s International Relations Office, includes the numbers of incoming and outgoing mobilities taken by students and staff per each academic year and the number of countries to which said mobilities are executed.

### Data analysis

We present the analysed variables as numbers and percentages for the whole university or medians and interquartile ranges (IQRs) for individual constituents.

Then, we used an interrupted time series analysis (ITSA) to evaluate the effects of interventions or events over time [41], allowing us to detect shifts in level and trends in a dependent variable following the onset of the COVID-19 pandemic as an intervention/disruption. To check whether the model fits the data, we examined diagnostic plots, including the residuals, fitted values, and a Q-Q plot [42]. We also included the Durbin-Watson statistics, which looks for autocorrelation in the residuals of a regression model [43]. Its values range from 0 to 4, where values around 2 indicate no autocorrelation, and values below or above 2 suggest positive or negative autocorrelation, respectively [44]. We further calculated heteroskedasticity-robust standard errors to check whether variability in the data influenced the estimated standard errors [45]. We applied the augmented Dickey-Fuller test to assess whether the yearly averages of the outcome variable were non-stationarity [46].

We performed the ITSA using Python, version 3.8.19 (Python Software Foundation, Wilmington, Delaware, USA) and its ‘stats models’ module, and visualised graphs using its ‘matplotlib’ library.

In a separate analysis, we analysed the differences in medians for individual indicators over years using the non-parametric Friedman test, followed by the Conover *post-hoc* test [47]. We used Bonferroni correction for multiple comparisons, setting the *P*-value threshold at 0.005 for academic indicators, 0.007 for publication output indicators, 0.003 for financial indicators, and 0.008 for mobility indicators. These analyses were performed in JASP, version 0.19.3 (JASP Team).

## RESULTS

We noted no statistically significant differences for any indicators at the level of the whole university (**Table 1**, **Table 2**, **Table 3**, **Table 4**). When analysing the indicators were analysed as the change in medians from all university constituents (Figures S2–5 and Data S1 in the **Online Supplementary Document**), however, we observed fluctuations observed that were not temporally related to the onset of COVID-19 pandemic.

**Table 1:** Academic indicators for the University of Split from 2016/2017 to 2022/2023 academic year

Indicator	2016/2017	2017/2018	2018/2019	2019/2020	2020/2021	2021/2022	2022/2023
**Students and public funding support**							
Total number of students enrolled	19 249	19 287	18 883	18 883	18 928	18 571	18 396
Number of students, first enrolment*	4286	4139	4033	4177	4113	4166	3863
Number of students, senior years†	4145	4017	3829	3995	4407	3900	3618
Total number of students receiving public subsidy	8431	8156	7862	8171	8520	8066	7481
Total public subsidy amount in EUR	5 712 025	5 541 974	5 519 232	5 706 921	5 909 908	5 648 829	5 274 818
**Studying success**							
Number of students who gave up studies in the first study year	1154	864	936	740	937	1037	1004
Number of students who gave up studies in any study year	1882	1929	1667	1323	1633	1811	1527
Number of students who did not fulfil requirements for a higher study year (<42 ECTS points)	6134	8748	8492	7739	8378	8634	8622
Number of students who fulfilled requirements for a higher study year (>60 ECTS points)	3692	5773	5356	5900	5557	5078	4763
Number of students who graduated	3870	3747	3802	3937	3937	3595	3608

*Students enrolled for the first time in a bachelor's, master's, or professional programmes who are entitled to receive the state subsidy.

†Students who passed the first year of a bachelor's, master's, or professional programmes who are entitled to receive the state subsidy (achieved more than 55 ECTS credits in the previous year).

**Table 2:** Academic staff and scientific output of the University of Split from calendar year 2017 to 2022

Indicator	2017	2018	2019	2020	2021	2022†
University staff*						
Total number of academic staff	715.8	711.4	752.5	772.9	788.4	796.2
Number of academic staff (STEM + health)	384.1	380.7	405.4	421.3	427.8	440.1
Number of academic staff (SSH)	266.0	265.0	279.6	285.1	287.1	285.1
Number of academic staff (ART)	65.7	65.7	67.5	66.5	73.5	71.0
**Publishing output (journal articles)**						
Total number of publications‡	912.5	1004.0	1164.3	1096.1	1166.8	1159.6
Number of publications in STEM + health journals	764.5	819.0	971.3	863.1	894.1	952.6
Number of publications in SH journals	148.0	175.0	186.0	221.0	267.7	179.0
Number of publications in ART journals	0.00	10.0	7.0	12.0	5.0	28.0

ART – art, SSH – social sciences and humanities, STEM – science, technology, engineering, and mathematics

*The number of employees, expressed as a decimal number, indicates that some faculties/departments employ their academic staff part-time.

†The last date for the extraction of data on scientific productivity was 2022 due to the time lag regarding the procedure of indexation of scientific publications in the Web of Science and Scopus databases for which the University Library is in charge. Only by June 2025, the University will have obtained the comprehensive data on scientific productivity for 2023.

‡Some articles had several authors from different faculties. According to the university procedure, a single article is divided into corresponding parts to provide adequate information on scientific productivity per institution. Therefore, the number of articles per faculty/department was expressed as decimal numbers. The decimal digits in summary numbers for the University level mean we did not include all published articles in our analysis. We excluded scientific papers exclusively linked to ongoing projects and without connection to observed faculty or departments.

**Table 3:** Financial indicators for the University of Split from business year 2017 to 2023, expressed in EUR

Indicator	2017	2018	2019	2020	2021	2022	2023
Revenues*							
Total revenues	77 704 813	76 197 260	89 640 582	79 294 210	86 146 209	91 306 039	95 149 630
Business revenues	65 820 880	72 382 381	87 650 169	78 184 334	85 063 530	89 311 340	94 085 909
Revenues from tuition fees	11 474 912	12 213 572	11 918 673	11 898 168	10 980 726	11 844 220	11 593 116
Revenues from market operations	4 355 094	4 267 168	4 322 018	3 216 606	3 932 102	4 171 052	5 415 068
Revenues from the sale of non-financial assets	440 179	2 677 950	892 984	5607	18 242	932 917	1938
Revenues from financial assets and borrowing	11 443 754	1 136 930	1 097 428	1 104 269	1 064 437	1 061 782	1 061 782
Uncollected revenues	1 754 529	1 673 447	1 683 139	2 122 521	2 664 711	2 868 301	2 593 304
**Expenditures†**							
Total expenditure	76 579 786	75 240 336	84 802 792	81 740 509	85 098 904	88 122 776	93 819 225
Business expenditures	58 778 084	63 437 316	67 164 877	65 603 615	72 203 060	79 199 812	87 189 281
Expenditures for business trips	1 186 027	1 336 816	1 554 826	418 416	659 277	1 705 730	2 023 184
Expenditure for purchase of non-financial assets	16 640 432	4 497 110	12 808 053	15 075 111	11 417 618	7 861 182	6 708 508
Expenditures for loan repayments	1 161 270	7 305 910	4 829 862	1 061 782	1 478 226	1 061 782	3 180
**Business result‡**							
Surplus of revenues available in the next period	7 917 439	8 076 016	8 799 841	8 493 211	9 165 793	10 728 635	12 129 872
Deficit of revenues to cover the next period	641 885	73 131	790 488	1 863 999	1 819 660	640 835	564 566
Average number of employees§	1793	1889	1933	1967	2009	2056	2022
**Financial indicators**							
Share (fraction) of public subsidies in total revenues¶	0.07	0.07	0.06	0.07	0.07	0.06	0.06
Annual surplus/deficit as a fraction of total revenues║	0.01	0.01	0.05	−0.03	0.01	0.03	0.01

*Explanation of indicators: revenues – the amount of money generated from normal business operations before any expenses are deducted.

†Explanation of indicators: business expenditures – the amount of money invested or spent for business operations.

‡Explanation of indicators: business result – the financial outcome of economic activities over the fiscal year period. It can be positive (surplus) when total revenues exceed total expenses or negative (deficit) when total expenses exceed total revenues.

§The average number of employees in the institution is calculated using the figures from the beginning and end of the reporting period (reported as such in official financial reports).

¶This measure reflects the extent to which an institution relies on government appropriations, which are typically the most stable source of revenue for public universities. Consistent proportions across years suggest that the University remained insulated from market-related shocks during the pandemic period.

║This is an indicator of financial stability. This metric shows the institution’s operating balance after all expenses, relative to its total income, and is commonly used to evaluate fiscal sustainability in publicly funded higher education systems. Stable or slightly positive ratios suggest consistent budget management and resilience to external shocks.

**Table 4:** Outgoing and incoming mobilities of staff and students from academic year 2016/2017 to 2022/2023

Indicator	2016/2017	2017/2018	2018/2019	2019/2020	2020/2021	2021/2022	2022/2023
Number of outgoing countries	58	59	59	53	41	57	58
Outgoing student mobility	235	239	285	212	196	249	317
Outgoing staff mobility	125	168	222	160	71	305	288
Number of incoming countries	42	42	55	37	27	55	46
Incoming student mobility	213	205	278	207	213	372	417
Incoming staff mobility	96	95	269	43	74	209	163

### Academic indicators

Among academic indicators (**Table 1**; Figure S1 in the **Online Supplementary Document**), there was no significant temporal change in the number of first-year students, the total number of enrolled students, or the total number of students who graduated. There were significant temporal changes in the median number of students advancing to a senior year, students who gave up studying in the first or any other study year, and students who did or did not fulfil the requirements for the higher study year. However, these changes were not related to the COVID-19 pandemic, but varied across university constituents (**Online Supplementary Document**). Consistent with this interpretation, visual inspection of faculty-level trajectories showed no clear or synchronised shift during the main pandemic period (2020–21). Significant changes were observed at the faculty (constituent) level for the following indicators (*P* < 0.001, Friedman test): the median number of students who gave up studies in the first study year, those who did not fulfil requirements for higher study year, and those who fulfilled requirements for higher study year. A *post-hoc* analysis showed that the observed differences among the study yeas were not related to the onset of the pandemic (**Online Supplementary Document**).

### Publication output indicators

With regard to the indicators related to the number of academic staff and their publication output, there were no significant differences over the years for the staff in SSH and ART disciplines, with unchanged or increasing number of academic staff for STEM + health disciplines (**Table 2**; Figure S2 in the **Online Supplementary Document**). The same was true for the number of articles published in indexed journals.

Descriptive patterns differed among faculties, with significant changes over the years + for the median of the total number of academic staff (Friedman test, *P* < 0.001), mostly due to the increase in the number of academic staff at STEM + health university components. A *post-hoc* analysis showed that the observed differences among the study years were not related to the onset of the pandemic (**Online Supplementary Document**).

### Financial indicators

The university’s overall financial indicators remained stable throughout the pandemic, with no significant disruptions in observed financial metrics across the years (**Table 3**; Figure S4 in the **Online Supplementary Document**). Despite the COVID-19 pandemic, faculties with high tuition income, such as the School of Medicine and Faculty of Economics, Business, and Tourism, reported consistent revenue streams during the pandemic, supported by robust enrolment figures (**Online Supplementary Document**). There was no change in the average number of employees, which registered a small, but consistent increase over the years. The revenues generated from market operations at the University of Split fluctuated between 2017 and 2023, with a notable decline during the pandemic years 2020 and 2021, and a subsequent recovery in 2022 and 2023.

The share of public subsidies in total revenue remained within a narrow interval across the study period at the University level (5.54–7.35%), and showed a significant decrease over the years. The annual surplus/deficit ratio ranged from –3.1% to +5.4% during the 2017–23 period, staying within the expected operational range.

At the level of university constituents, we noted significant differences (Friedman test, *P* < 0.001) + for the median total revenues, median business revenues, and the share of public subsidies in total revenue, but the *post-hoc* test did not show that these differences were related to the onset of the pandemic (**Online Supplementary Document**).

### Mobilities of staff and students

With regard to mobility (**Table 4**; Figure S4 in the **Online Supplementary Document**), outgoing student mobility decreased from 235 at the level of the whole university in the academic year 2018/2019 to 196 in the academic year 2020/2021, reflecting a decline of 17%. Similarly, outgoing staff mobility dropped by 43% from 125 to 71 during the same period. Incoming mobility trends mirrored these declines, with reductions in both incoming students and staff in the 2019/2020 and 2020/2021 academic years. However, recovery began in 2021/2022, and by 2022/2023, mobility figures for both students and staff surpassed pre-pandemic levels, *e.g.* outgoing student mobility increased to 317, while incoming student mobility rose to 417 in 2022/2023. The differences in median numbers for all mobility indicators were significant at the level of individual university constituents (Friedman test, *P* < 0.001) (**Online Supplementary Document**).

### ITSA

All indicators – academic, research, business and mobility – did not show significant changes in the amount or the slope of change in the ITSA after the start of the COVID-19 pandemic (**Table 5**). Regarding model fit, fitted values remained nearly constant across years, while the residuals were widely scattered without a temporal pattern. Q-Q plots showed clear deviations from the normal distribution. The Durbin-Watson statistic was close to 2 for all models, indicating there was no autocorrelation in the residuals; however, due to a small number of time points, this test has limited power. The augmented Dickey-Fuller test suggested that the yearly series was not stationary, although this conclusion should also be interpreted with caution due to a small number of observations. Applying heteroskedasticity-robust standard errors showed that the estimated effects and their *P*-values remained non-significant.

## DISCUSSION

The University of Split has shown resilience during the COVID-19 pandemic, with key academic, research and financial indicators quickly stabilising after initial disruptions.

Financial stability and unharmed financial indicators observed here contrast the findings of other studies on the pandemic’s economic impact on HEIs elsewhere in the world [27,48]. Unlike publicly funded European universities, Australian universities faced significant financial challenges during the COVID-19 pandemic, primarily due to their dependence on international student enrolment, which decreased markedly after border closures [6]. In comparison, the relative economic stability at the University of Split may be attributed to its publicly funded structure and ongoing support from the relevant Ministry. State-funded European HEIs were reported to have better short-term financial status during the COVID-19 pandemic [28]. Our study extends these findings to show that publicly funded universities may be less prone to long-term pandemic-related financial risks.

We observed no significant disruptions in the University of Split's overall scientific output during the observed period. In fact, the number of scientific publications related to STEM + health discipline slightly increased during the pandemic, while that of SSH publications decreased. A significant increase in published papers, particularly COVID-19–related manuscripts, was also reported in other academic settings, primarily within biomedical and health-science journals, which implemented accelerated editorial processes for COVID-19-related submissions [26]. The literature demonstrates varied effects of the COVID-19 pandemic on research productivity, with several studies documenting a decline among financial economists and academics [20,21,49].

These findings also suggest that, in its response to the COVID-19 pandemic, the University of Split exhibited the characteristic of an antifragile system, described by Nassim Taleb as a system with the capacity to gain from stressors and become more capable in managing future risks or shocks [50,51]. Mackey and colleagues [52] highlighted the importance of academic resilience in the context of teaching and learning continuity following the 2011 New Zealand earthquake, demonstrating that blended learning approaches can support educational processes during periods of disruption. The authors used the example of the earthquake that struck New Zealand in 2011, when the country faced challenges comparable to those that emerged during the COVID-19 pandemic, when traditional teaching methods were disrupted due to campus closures.

A factor that may have contributed to the resilience of the University of Split is the experience of its leadership during previous man-made crises, such as the Homeland War from 1991 to 1995 [53]. Croatia and Bosnia and Herzegovina’s experience with the War was also contextualised in the antifragility of their healthcare systems in responding to the pandemic [54]. Studies have emphasised the vital role of an innovative culture in institutional adaptability [55,56]. Leadership is a crucial element in academic resilience and crisis management. Effective responses rely on having leaders or crisis teams with relevant expertise [57]. Incorporating crisis leadership into recruitment and professional development enhances preparedness and ongoing stability [55,58]. The COVID-19 crisis is not unprecedented: earthquakes, wars, and other disasters have long served as opportunities for institutional learning and growth [52–54,59].

The literature suggests that, during post-crisis periods, universities may either return to business-as-usual practices or apply lessons learned during crises, such as the COVID-19 pandemic, to support institutional resilience and longer-term improvements [60]. Universities worldwide successfully maintained their teaching functions during the pandemic; however, the extent of damage varied across higher education systems, depending on institutional characteristics and funding models [61].

The decline in mobility during 2020–2021 reflects broader European trends, as Erasmus+ and other mobility programs were temporarily suspended or reorganised across Europe in response to the European Commission’s pandemic guidance issued in March 2020 [62]. The subsequent recovery in mobility at the University of Split should be seen in the context of Europe-wide reopening and policy coordination.

It has to be emphasised that university constituents vary in size, disciplinary profiles, funding arrangements, and publication cultures, and that such differences were evident in the descriptive plots. However, the goal of this study was to characterise institution-wide longitudinal trends, rather than to statistically assess causal differences between individual faculties.

To the best of our knowledge, our study is the first to assess all aspects of the university response to a pandemic and its impact on the academic, research, and financial indicators. Comprehensive, multidomain, longitudinal institutional studies using administrative data remain rare, especially in publicly funded higher education systems in Central, Eastern, and Southeastern Europe. Most published research on COVID-19 in higher education focuses on students’ or staff experiences, mental health, online teaching, or short-term surveys, while comprehensive university analyses that evaluate academic, financial, mobility, and research indicators together are nearly absent from the literature.

### Limitations

The main limitation of this study is its focus on the experience of a single university; it had no formal control group, as comparable multi-year administrative datasets from other Croatian universities were not publicly accessible in a harmonised format. This limits external validity and prevents direct counterfactual evaluation. Croatia has eight other public universities of different sizes and histories; our results can therefore be partially generalised to these institutions, and, in a more limited sense, to other publicly funded universities in countries with similar socioeconomic characteristics, particularly in Central, Eastern, and Southeastern Europe [63]. Future research could analyse the various impacts of disease outbreaks and other crises on faculty demographics (students and academic staff), interdisciplinary collaborations, and long-term institutional policies.

The main methodological limitation is the small number of time points (seven yearly observations), which substantially reduces the statistical power of the ITSA. Diagnostic tests such as the Durbin-Watson statistic, Q-Q plots, and the augmented Dickey-Fuller test also have limited reliability with sparse data, and the residuals showed deviations from normality. Because of this, the results of our ITSA should be interpreted as exploratory and descriptive, rather than as clear evidence that the pandemic had no impact on the University.

We assessed scientific output solely based on publications indexed in Web of Science and Scopus, ensuring international comparability, but excluding locally indexed journals, conference papers, and other types of literature. More detailed bibliometric indicators (*e.g.* impact factors, citation counts, field-normalised metrics) were not available in the administrative data set and therefore could not be included in the analysis.

Our results may serve as reference point for future research on institutional preparedness and adaptability in higher education, and for broader discussions on the role of institutional leadership during crises.

## CONCLUSIONS

Our study showed that the University of Split, as a whole and across its faculties and departments, did not experience measurable disruptions in key academic and financial indicators during the recent health crisis. We identified no significant disruptions to its operations, reflecting institutional resilience and stability during the crisis. These findings offer insights for enhancing preparedness and adaptability in HEIs and provide descriptive evidence of stability at the University of Split during the COVID-19 pandemic.

This study also underscores the importance of introducing resilient systems in higher education to mitigate risks, create opportunities during crises, and foster a better understanding of institutional resilience globally. During the observed period, the university budget remained intact, while internationalisation remained relatively low. However, future threats could continue to expose HEIs to significant risks, so it is advisable to reassess regulatory, normative, and current practices to identify weak points and to introduce a contingency plan for the higher education system as a whole.

## Additional material


Online Supplementary Document
