# Supplementary material for: Impact of the COVID-19 pandemic on university performance: a retrospective follow-up study of the University of Split, Croatia
Source: J Glob Health. 2026 Jan 12;16:04017. doi: 10.7189/jogh.16.04017 (PMC12794370; doi:10.7189/jogh.16.04017)

Supplement to: Hrga J, Mijatović A, Ljutić D, Marušić A. Impact of the COVID-19 pandemic on university performance: a retrospective follow-up study of the University of Split, Croatia. J Glob Health. 2026;16:04017.

Table S1. Academic indicators for the constituents of the University of Split from 2016/2017 to 2022/2023 academic year

**Indicator**	**2016/2017**	**2017/2018**	**2018/2019**	**2019/2020**	**2020/2021**	**2021/2022**	**2022/2023**
**Faculty of Economics, Business, and Tourism**							
No. of students, first enrollment	778	746	741	762	760	742	657
No. of students, senior years	494	499	454	444	657	330	306
Total no. of students receiving public subsidy	1,272	1,245	1,195	1,206	1,417	1,072	963
Total public subsidy amount (Eur)	698,069	683,854	656,593	661,358	775,964	585,042	524,746
Total No. students enrolled	2,815	2,750	2,698	2,622	2,665	2,449	2,425
No. students who graduated	707	695	615	560	632	406	555
No. students who fulfilled requirements for higher study year (>60 ECTS points)	339	729	513	861	510	401	323
No. students who did not fulfill requirements for higher study year (<42 ECTS points)	648	1,252	1,353	921	1,481	1,428	1,539
No. students who gave up studies in first study year	111	206	265	183	281	260	252
No. students who gave up studies at any time	472	570	564	303	592	715	372
**Faculty of Electrical Engineering, Mechanical Engineering and Naval Architecture**							
No. of students, first enrollment	793	726	705	775	714	659	663
No. of students, senior years	525	514	515	493	538	453	382
Total no. of students receiving public subsidy	1,318	1,240	1,220	1,268	1,252	1,112	1,045
Total public subsidy amount (Eur)	964,789	908,806	954,091	984,193	970,137	864,981	815,691
Total No. students enrolled	2,351	2,406	2,386	2,396	2,382	2,260	2,198
No. students who graduated	540	502	559	563	551	520	453
No. students who fulfilled requirements for higher study year (>60 ECTS points)	417	732	751	762	716	588	543
No. students who did not fulfill requirements for higher study year (<42 ECTS points)	847	956	1,004	1,025	1,065	1,129	1,121
No. students who gave up studies in first study year	329	116	142	149	208	250	155
No. students who gave up studies at any time	397	394	340	304	257	296	171

**Faculty of Civil Engineering, Architecture and Geodesy**							
No. of students, first enrollment	265	291	296	287	268	300	268
No. of students, senior years	206	210	239	248	271	226	212
Total no. of students receiving public subsidy	471	501	535	535	539	526	480
Total public subsidy amount (Eur)	345,929	368,704	419,709	420,784	421,461	411,587	376,271
Total No. students enrolled	909	965	995	992	1,001	1,036	1,066
No. students who graduated	214	211	227	225	224	208	224
No. students who fulfilled requirements for higher study year (>60 ECTS points)	169	310	297	326	271	242	250
No. students who did not fulfill requirements for higher study year (<42 ECTS points)	208	398	428	401	468	533	552
No. students who gave up studies in first study year	62	31	36	25	36	38	23
No. students who gave up studies at any time	72	102	95	73	83	77	84
**Faculty of Humanities and Social Sciences**							
No. of students, first enrollment	294	336	335,5	342,5	322	342	310
No. of students, senior years	573	513	399	576,5	554	516	487
Total no. of students receiving public subsidy	867	849	735	919	876	858	797
Total public subsidy amount (Eur)	508,149	497,684	419,185	524,481	499,940	489,668	454,856
Total No. students enrolled	1,178	1,218	1,370	1,435	1,433	1,420	1,489
No. students who graduated	342	301	343	361	385	329	317
No. students who fulfilled requirements for higher study year (>60 ECTS points)	432	709	680	728	670	661	609
No. students who did not fulfill requirements for higher study year (<42 ECTS points)	67	292	316	315	333	359	427
No. students who gave up studies in first study year	35	34	38	42	42	56	49
No. students who gave up studies at any time	54	64	80	68	68	78	82
**Catholic Faculty of Theology**							
No. of students, first enrollment	56	58	41	30	31	35	34
No. of students, senior years	115	84	93	89	76	67	65
Total no. of students receiving public subsidy	171	142	134	119	107	102	99
Total public subsidy amount (Eur)	102,130	84,810	76,475	67,914	61,066	58,212	56,500
Total No. students enrolled	267	237	245	234	200	182	163
No. students who graduated	30	38	32	49	46	36	31

No. students who fulfilled requirements for higher study year (>60 ECTS points)	65	81	80	81	73	74	65
No. students who did not fulfill requirements for higher study year (<42 ECTS points)	14	70	62	75	63	42	31
No. students who gave up studies in first study year	5	1	10	3	5	0	5
No. students who gave up studies at any time	32	27	29	22	20	5	22
**Faculty of Chemistry and Technology**							
No. of students, first enrollment	224	188	204	210	173	148	138
No. of students, senior years	198	132	141	130	130	128	79
Total no. of students receiving public subsidy	422	320	345	340	303	276	217
Total public subsidy amount (Eur)	322,915	241,078	272,467	269,918	240,812	219,670	172,686
Total No. students enrolled	660	605	568	555	550	513	459
No. students who graduated	127	125	132	129	128	145	122
No. students who fulfilled requirements for higher study year (>60 ECTS points)	87	202	143	109	133	91	86
No. students who did not fulfill requirements for higher study year (<42 ECTS points)	75	207	233	235	255	271	245
No. students who gave up studies in first study year	78	46	52	45	57	52	49
No. students who gave up studies at any time	82	94	80	67	65	60	52
**Faculty of Kinesiology**							
No. of students, first enrollment	133	114	106	126	114	122	102
No. of students, senior years	118	136	121	118	144	118	108
Total no. of students receiving public subsidy	251	250	227	244	258	240	210
Total public subsidy amount (Eur)	143,248	142,677	129,551	139,253	147,243	136,970	119,849
Total No. students enrolled	809	828	778	831	838	849	767
No. students who graduated	168	168	184	187	146	195	177
No. students who fulfilled requirements for higher study year (>60 ECTS points)	203	215	192	89	230	213	257
No. students who did not fulfill requirements for higher study year (<42 ECTS points)	330	390	389	332	366	360	291
No. students who gave up studies in first study year	15	14	17	7	11	29	49
No. students who gave up studies at any time	28	28	42	17	18	51	146
**University of Split, School of Medicine**							
No. of students, first enrollment	123	123	134	151	128	148	152

No. of students, senior years	479	595	595	613	629	604	570
Total no. of students receiving public subsidy	602	718	729	764	757	752	722
Total public subsidy amount (Eur)	479,395	571,770	580,530	608,401	602,827	598,845	574,957
Total No. students enrolled	1,158	1,347	1,522	1,655	1,735	1,723	1,715
No. students who graduated	127	164	153	186	219	276	230
No. students who fulfilled requirements for higher study year (>60 ECTS points)	638	843	889	940	894	911	839
No. students who did not fulfill requirements for higher study year (<42 ECTS points)	117	170	146	135	206	207	204
No. students who gave up studies in first study year	15	5	9	7	12	18	12
No. students who gave up studies at any time	49	33	30	32	35	44	36
**Faculty of Maritime Studies**							
No. of students, first enrollment	320	302	300	304	299	274	256
No. of students, senior years	298	266	238	218	203	194	161
Total no. of students receiving public subsidy	618	568	538	522	502	468	417
Total public subsidy amount (Eur)	459,327	422,165	428,429	415,688	399,761	372,686	332,074
Total No. students enrolled	1,546	1,506	1,436	1,430	1,394	1,372	1,272
No. students who graduated	304	278	225	295	198	222	211
No. students who fulfilled requirements for higher study year (>60 ECTS points)	207	308	293	260	223	221	170
No. students who did not fulfil requirements for higher study year (<42 ECTS points)	442	820	784	803	787	814	796
No. students who gave up studies in first study year	104	32	37	32	47	42	62
No. students who gave up studies at any time	157	45	58	45	48	49	69
**Faculty of Law**							
No. of students, first enrollment	353	286	273	258	255	207	162
No. of students, senior years	262	208	162	150	147	220	150
Total no. of students receiving public subsidy	615	494	435	408	402	427	312
Total public subsidy amount (Eur)	334,023	268,870	237,826	221,222	218,037	233,340	171,850
Total No. students enrolled	3,277	3,035	2,594	2,449	2,386	2,134	2,086
No. students who graduated	390	366	319	301	385	278	250
No. students who fulfilled requirements for higher study year (>60 ECTS points)	272	486	329	346	390	283	261
No. students who did not fulfil requirements for higher study year (<42 ECTS points)	1,983	2,007	1,734	1,658	1,501	1,426	1,289

No. students who gave up studies in first study year	119	107	79	57	63	52	42
No. students who gave up studies at any time	182	165	0	134	203	80	75
**Faculty of Science**							
No. of students, first enrollment	276	262	234	237	305	283	295
No. of students, senior years	158	166	191	185	188	216	163
Total no. of students receiving public subsidy	434	428	425	422	493	499	458
Total public subsidy amount (Eur)	343,274	338,337	338,443	336,054	392,594	397,372	364,724
Total No. students enrolled	854	883	895	922	959	1,014	1,067
No. students who graduated	200	168	158	245	186	213	216
No. students who fulfilled requirements for higher study year (>60 ECTS points)	111	189	171	213	211	191	182
No. students who did not fulfill requirements for higher study year (<42 ECTS points)	181	395	403	427	435	485	545
No. students who gave up studies in first study year	94	41	40	41	43	46	42
No. students who gave up studies at any time	114	83	94	83	90	91	93
**University Department of Professional Studies**							
No. of students, first enrollment	384	407	386	396	450	449	413
No. of students, senior years	259	233	214	259	353	343	242
Total no. of students receiving public subsidy	643	640	600	655	803	792	655
Total public subsidy amount (Eur)	365,797	367,257	350,946	383,157	473,117	469,387	388,918
Total No. students enrolled	2,274	2,363	2,258	2,163	2,131	2,142	2,105
No. students who graduated	408	439	515	512	462	431	387
No. students who fulfilled requirements for higher study year (>60 ECTS points)	281	466	485	598	568	472	474
No. students who did not fulfil requirements for higher study year (<42 ECTS points)	965	1,412	1,245	1,019	1,027	1,115	1,091
No. students who gave up studies in first study year	161	210	191	140	112	153	243
No. students who gave up studies at any time	187	296	229	158	130	207	286
**University Department of Marine Studies**							
No. of students, first enrollment	41	55	46	41	33	40	46
No. of students, senior years	60	58	52	48	65	51	43
Total no. of students receiving public subsidy	101	113	98	89	98	91	89
Total public subsidy amount (Eur)	76,395	84,943	78,041	70,874	78,041	72,467	70,874

Total No. students enrolled	190	153	154	180	150	146	148
No. students who graduated	55	43	41	37	41	43	40
No. students who fulfilled requirements for higher study year (>60 ECTS points)	48	60	56	60	55	47	55
No. students who did not fulfil requirements for higher study year (<42 ECTS points)	27	51	63	56	61	74	65
No. students who gave up studies in first study year	2	5	4	4	3	2	4
No. students who gave up studies at any time	5	12	7	7	3	4	6
University Department of Health Studies							
No. of students, first enrollment	100	85	90	84	87	126	105
No. of students, senior years	166	171	173	188	198	190	208
Total no. of students receiving public subsidy	266	256	263	272	285	316	313
Total public subsidy amount (Eur)	211,826	203,862	209,437	216,604	226,956	251,642	249,254
Total No. students enrolled	462	462	471	493	548	656	708
No. students who graduated	134	100	136	136	157	141	216
No. students who fulfilled requirements for higher study year (>60 ECTS points)	228	207	203	226	305	326	332
No. students who did not fulfill requirements for higher study year (<42 ECTS points)	193	197	204	216	195	232	216
No. students who gave up studies in first study year	14	10	10	2	12	18	8
No. students who gave up studies at any time	17	10	12	4	12	30	11
**University Department for Forensic Sciences**							
No. of students, first enrollment	39	39	32	58	65	162	141
No. of students, senior years	15	44	37	38	56	67	128
Total no. of students receiving public subsidy	54	83	69	96	121	229	269
Total public subsidy amount (Eur)	32,252	49,572	54,947	76,448	96,357	182,361	214,215
Total No. students enrolled	87	101	100	122	154	270	338
No. students who graduated	25	31	34	39	45	57	58
No. students who fulfilled requirements for higher study year (>60 ECTS points)	39	6	42	67	84	124	122
No. students who did not fulfill requirements for higher study year (<42 ECTS points)	8	21	29	28	28	59	102
No. students who gave up studies in first study year	3	4	0	0	1	14	5
No. students who gave up studies at any time	3	6	1	0	1	15	9

**Art Academy**							
No. of students, first enrollment	107	121	109	115	109	129	77
No. of students, senior years	219	188	205	197	198	177	191
Total no. of students receiving public subsidy	326	309	314	312	307	306	268
Total public subsidy amount (Eur)	324,507	307,585	312,562	310,571	305,594	304,599	266,773
Total No. students enrolled	412	428	413	404	402	405	390
No. students who graduated	99	118	129	112	132	95	121
No. students who fulfilled requirements for higher study year (>60 ECTS points)	156	230	232	234	224	233	195
No. students who did not fulfill requirements for higher study year (<42 ECTS points)	29	110	99	93	107	100	108
No. students who gave up studies in first study year	7	2	6	3	4	7	4
No. students who gave up studies at any time	31	0	6	6	8	9	13

Table S2. Academic staff and scientific output of the University of Split from 2017-2022 calendar year

Indicator	2017	2018	2019	2020	2021	2022
**Faculty of Economics, Business, and Tourism**						
Total No. of academic staff	68.00	68.00	72.80	71.80	71.80	69.80
No. of academic staff (STEM+health)	0.00	0.00	0.00	0.00	0.00	0.00
No. of academic staff (SH)	68.00	68.00	72.80	71.80	71.80	69.80
No. of academic staff (ART)	0.00	0.00	0.00	0.00	0.00	0.00
No. of publications in STEM+health journals	2.40	3.50	7.50	6.33	14.50	6.20
No. of publications in SH journals	54.50	50.00	44.67	59.83	92.50	51.33
No. of publications in ART journals	0.00	0.00	0.00	0.00	0.00	0.00
**Faculty of Electrical Engineering, Mechanical Engineering and Naval Architecture**						
Total No. of academic staff	102.00	102.00	105.00	104.00	104.00	104.00
No. of academic staff (STEM+health)	100.00	100.00	103.00	102.00	102.00	102.00
No. of academic staff (SH)	2.00	2.00	2.00	2.00	2.00	2.00
No. of academic staff (ART)	0.00	0.00	0.00	0.00	0.00	0.00
No. of publications in STEM+health journals	223.72	271.71	355.95	254.41	196.91	193.30
No. of publications in SH journals	3.00	3.33	2.25	4.67	0.50	3.00
No. of publications in ART journals	0.00	0.00	0.00	0.00	0.00	1.00
**Faculty of Civil Engineering, Architecture and Geodesy**						
Total No. of academic staff	60.75	60.75	63.50	64.50	63.50	65.00
No. of academic staff (STEM+health)	52.00	52.00	54.00	55.00	54.00	56.00
No. of academic staff (SH)	1.00	1.00	0.00	0.00	0.00	0.00
No. of academic staff (ART)	7.75	7.75	9.50	9.50	9.50	9.00

No. of publications in STEM+health journals	43.92	54.00	67.29	48.38	59.58	59.59
No. of publications in SH journals	4.00	6.00	3.00	3.00	0.00	2.00
No. of publications in ART journals	0.00	0.00	2.00	2.00	1.00	3.00
**Faculty of Humanities and Social Sciences**						
Total No. of academic staff	79.00	79.00	90.35	94.00	93.00	96.00
No. of academic staff (STEM+health)	1.00	1.00	1.00	2.00	2.00	2.00
No. of academic staff (SH)	78.00	78.00	88.35	91.00	91.00	94.00
No. of academic staff (ART)	0.00	0.00	1.00	1.00	0.00	0.00
No. of publications in STEM+health journals	5.48	11.12	17.80	7.23	6.49	5.89
No. of publications in SH journals	40.37	37.75	55.67	54.37	81.14	47.17
No. of publications in ART journals	0.00	10.00	4.00	8.00	4.00	18.00
**University Department for Forensic Sciences**						
Total No. of academic staff	7.25	7.25	10.00	11.00	13.50	14.50
No. of academic staff (STEM+health)	2.25	2.25	2.00	2.50	3.50	3.50
No. of academic staff (SH)	5.00	5.00	8.00	8.50	10.00	11.00
No. of academic staff (ART)	0.00	0.00	0.00	0.00	0.00	0.00
No. of publications in STEM+health journals	4.92	6.59	7.60	5.67	9.15	9.02
No. of publications in SH journals	3.50	1.00	6.25	9.17	10.17	11.17
No. of publications in ART journals	0.00	0.00	0.00	0.00	0.00	0.00
**Catholic Faculty of Theology**						
Total No. of academic staff	19.00	19.00	21.00	21.30	20.30	21.30
No. of academic staff (STEM+health)	0.00	0.00	0.00	0.00	0.00	0.00
No. of academic staff (SH)	19.00	19.00	21.00	21.30	20.30	21.30
No. of academic staff (ART)	0.00	0.00	0.00	0.00	0.00	0.00
No. of publications in STEM+health journals	1.00	0.00	0.00	0.00	0.33	1.00
No. of publications in SH journals	6.00	11.00	19.00	21.20	15.50	6.00
No. of publications in ART journals	0.00	0.00	0.00	0.00	0.00	0.00
**Faculty of Chemistry and Technology**						
Total No. of academic staff	42.00	40.00	48.00	49.50	48.50	50.00
No. of academic staff (STEM+health)	42.00	40.00	48.00	49.50	48.50	50.00
No. of academic staff (SH)	0.00	0.00	0.00	0.00	0.00	0.00
No. of academic staff (ART)	0.00	0.00	0.00	0.00	0.00	0.00
No. of publications in STEM+health journals	36.04	41.91	41.30	55.64	54.98	63.70
No. of publications in SH journals	1.00	0.00	0.00	0.00	0.00	0.00
No. of publications in ART journals	0.00	0.00	0.00	0.00	0.00	0.00
**Faculty of Kinesiology**						
Total No. of academic staff	35.50	34.50	32.50	33.50	35.50	35.00
No. of academic staff (STEM+health)	1.50	1.50	2.00	1.50	2.00	2.00
No. of academic staff (SH)	34.00	33.00	30.50	32.00	33.50	33.00
No. of academic staff (ART)	0.00	0.00	0.00	0.00	0.00	0.00
No. of publications in STEM+health journals	74.42	78.42	59.50	48.78	46.00	54.23
No. of publications in SH journals	2.33	18.00	20.50	18.00	6.17	9.67
No. of publications in ART journals	0.00	0.00	0.00	0.00	0.00	0.00

**University of Split, School of Medicine**						
Total No. of academic staff	80.95	82.57	78.55	85.34	87.57	85.45
No. of academic staff (STEM+health)	80.95	82.57	78.55	85.34	87.57	85.45
No. of academic staff (SH)	0.00	0.00	0.00	0.00	0.00	0.00
No. of academic staff (ART)	0.00	0.00	0.00	0.00	0.00	0.00
No. of publications in STEM+health journals	250.07	197.71	254.36	282.76	308.55	360.02
No. of publications in SH journals	2.50	14.25	11.25	15.50	11.30	14.08
No. of publications in ART journals	0.00	0.00	0.00	0.00	0.00	0.00
**Faculty of Science**						
Total No. of academic staff	63.13	62.13	68.55	71.00	70.00	72.20
No. of academic staff (STEM+health)	58.13	57.13	63.55	66.50	65.50	67.70
No. of academic staff (SH)	5.00	5.00	5.00	4.50	4.50	4.50
No. of academic staff (ART)	0.00	0.00	0.00	0.00	0.00	0.00
No. of publications in STEM+health journals	81.26	99.67	101.36	89.93	86.62	99.98
No. of publications in SH journals	2.80	4.67	4.42	9.77	6.33	8.50
No. of publications in ART journals	0.00	0.00	0.00	0.00	0.00	0.00
**Faculty of Maritime Studies**						
Total No. of academic staff	34.00	32.00	39.75	40.75	44.75	48.00
No. of academic staff (STEM+health)	25.00	23.00	29.75	29.75	33.75	38.50
No. of academic staff (SH)	9.00	9.00	10.00	11.00	11.00	9.50
No. of academic staff (ART)	0.00	0.00	0.00	0.00	0.00	0.00
No. of publications in STEM+health journals	20.27	30.85	30.28	37.26	47.33	34.99
No. of publications in SH journals	5.00	5.00	2.50	3.83	20.83	4.00
No. of publications in ART journals	0.00	0.00	0.00	0.00	0.00	1.00
**Faculty of Law**						
Total No. of academic staff	38.00	38.00	35.00	34.00	33.00	31.00
No. of academic staff (STEM+health)	0.00	0.00	0.00	0.00	0.00	0.00
No. of academic staff (SH)	38.00	38.00	35.00	34.00	33.00	31.00
No. of academic staff (ART)	0.00	0.00	0.00	0.00	0.00	0.00
No. of publications in STEM+health journals	2.83	1.33	1.00	0.00	0.00	1.00
No. of publications in SH journals	8.00	14.00	9.00	12.83	13.00	14.00
No. of publications in ART journals	0.00	0.00	0.00	0.00	0.00	0.00
**University Department of Marine Studies**						
Total No. of academic staff	17.00	17.00	15.00	16.00	17.00	17.00
No. of academic staff (STEM+health)	17.00	17.00	15.00	16.00	17.00	17.00
No. of academic staff (SH)	0.00	0.00	0.00	0.00	0.00	0.00
No. of academic staff (ART)	0.00	0.00	0.00	0.00	0.00	0.00
No. of publications in STEM+health journals	12.33	17.61	21.54	21.94	31.22	37.98
No. of publications in SH journals	0.00	0.00	0.00	0.00	0.00	0.00
No. of publications in ART journals	0.00	0.00	0.00	0.00	0.00	0.00
**University Department of Health Studies**						
Total No. of academic staff	5.25	5.25	8.50	13.25	13.95	18.95
No. of academic staff (STEM+health)	4.25	4.25	8.50	11.25	10.95	15.95

No. of academic staff (SH)	1.00	1.00	0.00	2.00	3.00	3.00
No. of academic staff (ART)	0.00	0.00	0.00	0.00	0.00	0.00
No. of publications in STEM+health journals	1.83	1.99	2.48	3.35	30.62	23.71
No. of publications in SH journals	2.00	3.00	2.00	1.50	1.72	2.08
No. of publications in ART journals	0.00	0.00	0.00	0.00	0.00	0.00
**Art Academy**						
Total No. of academic staff	63.00	64.00	64.00	62.00	70.00	68.00
No. of academic staff (STEM+health)	0.00	0.00	0.00	0.00	0.00	0.00
No. of academic staff (SH)	5.00	6.00	7.00	6.00	6.00	6.00
No. of academic staff (ART)	58.00	58.00	57.00	56.00	64.00	62.00
No. of publications in STEM+health journals	0.00	1.00	0.00	0.00	1.00	0.00
No. of publications in SH journals	2.00	2.00	1.00	4.00	0.50	3.00
No. of publications in ART journals	0.00	0.00	1.00	2.00	0.00	5.00
**University Department of Professional Studies**						
Total No. of academic staff	0.00	0.00	0.00	0.00	0.00	0.00
No. of academic staff (STEM+health)	0.00	0.00	0.00	0.00	0.00	0.00
No. of academic staff (SH)	0.00	0.00	0.00	0.00	0.00	0.00
No. of academic staff (ART)	0.00	0.00	0.00	0.00	0.00	0.00
No. of publications in STEM+health journals	4.00	1.58	3.33	1.42	0.83	2.00
No. of publications in SH journals	11.00	5.00	4.50	3.33	8.00	3.00
No. of publications in ART journals	0.00	0.00	0.00	0.00	0.00	0.00

Table S3. Financial indicators for the University of Split from 2017 to 2023 business year (in Euro)

Indicator	2017	2018	2019	2020	2021	2022	2023
**Faculty of Economics, Business, and Tourism**							
Business revenues	5,207,421	5,538,255	5,531,636	5,161,525	5,862,487	6,025,191	6,463,633
Revenues from tuition fees	1,235,318	1,235,288	1,096,623	922,266	833,833	931,934	985,626
Revenues from market operations	245,008	299,614	374,304	156,482	115,992	230,150	298,137
Revenues from the sale of non-financial assets	499	499	499	500	484	573	437
Revenues from financial assets and borrowing	425,514	0	0	0	0	0	0
Total revenues	5,633,434	5,538,754	5,532,135	5,162,026	5,862,971	6,025,765	6,464,070
Uncollected revenues	178,404	32,835	10,543	5,005	3,584	5,288	21,623
Business expenditures	5,137,845	5,250,550	5,317,590	5,161,525	5,547,601	5,625,057	6,207,887
Expenditures for business trips	64,086	100,506	139,163	24,784	38,397	111,685	212,233
Expenditure for purchase of non-financial assets	34,750	104,486	97,712	197,320	195,114	115,653	97,930
Expenditures for loan repayments	0	0	0	0	0	0	0

Total expenditure	5,172,595	5,355,036	5,415,301	5,358,845	5,742,714	5,740,710	6,305,817
Surplus of revenues available in the next period	206,984	390,702	507,536	811,725	932,374	1,217,474	1,353,770
Deficit of revenues to cover the next period	0	0	0	0	0	0	0
Average No. of employees	119	121	124	126	127	127	124
Share of public subsidies in total revenues	0.12	0.12	0.12	0.13	0.13	0.10	0.08
Annual surplus/deficit as a proportion of total revenues	0.08	0.03	0.02	-0.04	0.02	0.05	0.02
**Faculty of Electrical Engineering, Mechanical Engineering and Naval Architecture**							
Business revenues	8,002,098	8,813,593	8,658,764	8,921,013	9,696,897	9,903,494	11,091,707
Revenues from tuition fees	1,143,852	1,181,040	980,149	884,943	804,394	693,318	744,662
Revenues from market operations	393,918	605,767	484,786	587,959	738,348	869,809	1,435,524
Revenues from the sale of non-financial assets	4,530	1,041	3,934	3,328	728	490	1,121
Revenues from financial assets and borrowing	1,061,782	1,061,782	1,061,782	1,061,782	1,061,782	1,061,782	1,061,782
Total revenues	9,068,411	9,876,417	9,724,480	9,986,123	10,759,408	10,965,767	12,154,610
Uncollected revenues	297,412	403,729	177,827	333,670	256,704	387,898	115,197
Business expenditures	7,520,059	8,162,460	8,654,609	8,026,463	8,733,340	9,720,480	10,145,092
Expenditures for business trips	226,396	253,993	283,733	51,997	87,975	263,799	305,466
Expenditure for purchase of non-financial assets	386,006	528,960	356,483	569,218	502,183	978,258	378,420
Expenditures for loan repayments	1,061,782	1,061,782	1,061,782	1,061,782	1,061,782	1,061,782	0
Total expenditure	8,967,848	9,753,202	10,072,875	9,657,463	10,297,306	11,760,520	10,523,511
Surplus of revenues available in the next period	900,346	1,023,561	675,167	1,003,827	1,465,929	671,176	2,302,274
Deficit of revenues to cover the next period	0	0	0	0	0	0	0
Average No. of employees	248	255	256	252	259	264	254
Share of public subsidies in total revenues	0.11	0.09	0.10	0.10	0.09	0.08	0.07
Annual surplus/deficit as a proportion of total revenues	0.01	0.01	-0.04	0.03	0.04	-0.07	0.13
**Faculty of Civil Engineering,**							

**Architecture and Geodesy**							
Business revenues	5,048,121	5,561,431	7,310,746	8,883,433	10,917,147	7,782,362	7,629,124
Revenues from tuition fees	437,077	423,658	447,900	371,483	362,130	340,304	423,575
Revenues from market operations	983,780	1,447,212	1,027,081	749,821	892,020	960,049	1,281,594
Revenues from the sale of non-financial assets	652	444	2,407	913	803	190	199
Revenues from financial assets and borrowing	2,247	75,147	35,646	42,487	0	0	0
Total revenues	5,051,020	5,637,022	7,348,800	8,926,833	10,917,950	7,782,552	7,629,323
Uncollected revenues	257,127	317,690	159,659	205,141	270,156	414,991	365,546
Business expenditures	4,827,145	5,512,050	5,311,444	5,241,516	6,076,177	6,224,236	6,942,379
Expenditures for business trips	113,836	103,445	123,790	32,673	64,600	127,648	132,770
Expenditure for purchase of non-financial assets	122,788	116,356	1,839,980	4,633,673	4,695,972	227,052	326,185
Expenditures for loan repayments	99,488	6,156	49,186	0	0	0	0
Total expenditure	5,049,421	5,634,562	7,200,610	9,875,189	10,772,150	6,451,289	7,268,564
Surplus of revenues available in the next period	176,368	178,829	327,018	0	0	847,264	1,207,983
Deficit of revenues to cover the next period	0	0	0	628,241	487,113	0	0
Average No. of employees	135	140	143	153	153	150	139
Share of public subsidies in total revenues	0.07	0.07	0.06	0.05	0.04	0.05	0.05
Annual surplus/deficit as a proportion of total revenues	0.00	0.00	0.02	-0.11	0.01	0.17	0.05
**Faculty of Humanities and Social Sciences**							
Business revenues	4,920,660	5,037,665	5,215,047	5,418,558	5,839,775	6,321,484	6,861,528
Revenues from tuition fees	177,012	190,213	209,207	189,914	163,650	180,418	236,612
Revenues from market operations	249,186	292,583	278,024	248,303	398,911	518,477	512,493
Revenues from the sale of non-financial assets	0	0	0	0	0	0	0
Revenues from financial assets and borrowing	0	0	0	0	0	0	0
Total revenues	4,920,660	5,037,665	5,215,047	5,418,558	5,839,775	6,321,484	6,861,528
Uncollected revenues	38,073	32,700	72,055	78,206	84,920	56,809	39,094
Business expenditures	4,783,828	5,013,781	5,017,648	5,132,156	5,531,867	6,053,530	6,735,828
Expenditures for business trips	89,404	73,278	101,012	53,144	35,339	72,913	131,286

Expenditure for purchase of non-financial assets	48,739	98,975	200,558	139,159	99,589	81,334	110,107
Expenditures for loan repayments	0	0	0	0	0	0	0
Total expenditure	4,832,568	5,112,756	5,218,206	5,271,315	5,631,456	6,134,863	6,845,936
Surplus of revenues available in the next period	602,913	522,723	519,564	666,806	857,144	1,099,288	1,128,941
Deficit of revenues to cover the next period	0	0	0	0	0	0	0
Average No. of employees	164	166	170	168	167	170	173
Share of public subsidies in total revenues	0.10	0.10	0.08	0.10	0.09	0.08	0.07
Annual surplus/deficit as a proportion of total revenues	0.02	-0.01	0.00	0.03	0.04	0.03	0.00
**Catholic Faculty of Theology**							
Business revenues	1,307,636	1,340,823	1,516,694	1,495,866	1,662,271	1,668,800	1,745,820
Revenues from tuition fees	31,706	36,805	43,604	44,254	38,117	36,677	42,339
Revenues from market operations	17,407	27,997	76,207	10,923	44,704	48,003	18,678
Revenues from the sale of non-financial assets	0	0	0	0	0	0	0
Revenues from financial assets and borrowing	0	0	0	0	0	0	0
Total revenues	1,307,636	1,340,823	1,516,694	1,495,866	1,662,271	1,668,800	1,745,820
Uncollected revenues	12,162	41,733	31,005	22,138	12,561	15,788	0
Business expenditures	1,289,468	1,360,480	1,432,030	1,480,047	1,619,123	1,666,273	1,715,096
Expenditures for business trips	2,662	1,109	3,525	447	7,907	18,147	22,201
Expenditure for purchase of non-financial assets	1,804	2,769	66,831	11,811	2,024	12,187	4,177
Expenditures for loan repayments	0	0	0	0	0	0	0
Total expenditure	1,291,272	1,363,249	1,498,861	1,491,858	1,621,147	1,678,460	1,719,273
Surplus of revenues available in the next period	0	0	0	0	0	0	26,547
Deficit of revenues to cover the next period	50,705	73,131	55,297	51,290	10,166	19,827	0
Average No. of employees	51	51	51	51	52	50	49
Share of public subsidies in total revenues	0.08	0.06	0.05	0.05	0.04	0.03	0.03
Annual surplus/deficit as a proportion of total revenues	0.01	-0.02	0.01	0.00	0.02	-0.01	0.02

**Faculty of Chemistry and Technology**							
Business revenues	2,955,685	3,020,175	3,027,355	3,200,768	3,331,238	3,476,320	3,745,588
Revenues from tuition fees	342,330	359,497	194,752	179,562	162,262	126,642	137,365
Revenues from market operations	53,793	28,118	54,785	58,637	46,666	57,501	65,761
Revenues from the sale of non-financial assets	1,340	549	504	199	48	9	70
Revenues from financial assets and borrowing	0	0	0	0	0	0	0
Total revenues	2,957,024	3,020,724	3,027,859	3,200,967	3,331,286	3,476,330	3,745,658
Uncollected revenues	105,315	99,144	67,900	59,679	35,148	48,885	38,833
Business expenditures	2,821,700	2,899,982	2,978,490	2,953,468	3,114,777	3,492,872	3,629,562
Expenditures for business trips	46,923	60,826	57,774	8,459	22,888	52,175	70,586
Expenditure for purchase of non-financial assets	196,362	120,489	59,106	78,283	124,746	78,784	53,119
Expenditures for loan repayments	0	0	0	0	0	0	0
Total expenditure	3,018,062	3,020,471	3,037,596	3,031,751	3,239,522	3,571,656	3,682,681
Surplus of revenues available in the next period	100,428	97,464	114,735	283,685	373,396	277,753	340,730
Deficit of revenues to cover the next period	0	0	0	0	0	0	0
Average No. of employees	101	103	103	102	101	104	104
Share of public subsidies in total revenues	0.11	0.08	0.09	0.08	0.07	0.06	0.05
Annual surplus/deficit as a proportion of total revenues	-0.02	0.00	0.00	0.05	0.03	-0.03	0.02
**Faculty of Kinesiology**							
Business revenues	2,204,865	2,271,671	2,294,837	2,393,725	2,468,423	2,664,516	2,791,935
Revenues from tuition fees	626,664	651,238	577,434	619,316	574,542	475,078	455,623
Revenues from market operations	75,140	62,999	58,845	21,626	40,318	39,149	34,256
Revenues from the sale of non-financial assets	0	0	0	0	0	0	0
Revenues from financial assets and borrowing	0	0	0	0	0	0	0
Total revenues	2,204,865	2,271,671	2,294,837	2,393,725	2,468,423	2,664,516	2,791,935
Uncollected revenues	94,888	71,969	48,680	51,805	48,663	48,999	39,134
Business expenditures	2,225,104	2,323,674	2,241,226	2,098,733	2,223,441	2,628,982	2,792,686
Expenditures for business trips	54,488	62,764	74,588	21,665	26,229	57,167	76,385

Expenditure for purchase of non-financial assets	190,281	40,419	34,604	37,513	204,222	177,951	81,611
Expenditures for loan repayments	0	0	0	0	0	0	0
Total expenditure	2,415,384	2,364,092	2,275,831	2,136,246	2,427,663	2,806,932	2,792,553
Surplus of revenues available in the next period	241,496	148,194	167,182	424,661	465,421	322,235	240,947
Deficit of revenues to cover the next period	0	0	0	0	0	0	0
Average No. of employees	64	64	60	60	61	63	60
Share of public subsidies in total revenues	0.06	0.06	0.06	0.06	0.06	0.05	0.04
Annual surplus/deficit as a proportion of total revenues	-0.10	-0.04	0.01	0.11	0.02	-0.05	0.00
**University of Split, School of Medicine**							
Business revenues	7,389,098	8,149,273	8,098,319	9,304,134	10,287,485	10,873,801	11,748,060
Revenues from tuition fees	2,908,444	3,050,204	3,118,568	3,178,201	3,721,882	3,340,187	3,312,698
Revenues from market operations	224,381	396,089	436,865	359,333	473,961	377,242	732,442
Revenues from the sale of non-financial assets	0	0	0	0	0	0	0
Revenues from financial assets and borrowing	0	0	0	0	0	0	0
Total revenues	7,389,098	8,149,273	8,098,319	9,304,134	10,287,485	10,873,801	11,748,060
Uncollected revenues	62,240	189,537	273,664	277,759	392,235	208,979	305,408
Business expenditures	6,645,841	7,551,181	8,387,126	8,132,331	9,818,087	10,693,012	10,832,329
Expenditures for business trips	144,746	189,531	227,137	48,901	62,906	267,161	288,148
Expenditure for purchase of non-financial assets	316,984	524,882	712,980	508,266	519,825	689,052	282,532
Expenditures for loan repayments	0	0	0	0	0	0	0
Total expenditure	6,962,825	8,076,063	9,100,106	8,640,597	10,337,913	11,382,065	11,114,861
Surplus of revenues available in the next period	220,714	293,924	0	0	0	0	0
Deficit of revenues to cover the next period	0	0	735,190	76,384	85,589	621,009	18,089
Average No. of employees	251	274	276	283	294	295	285
Share of public subsidies in total revenues	0.06	0.07	0.07	0.07	0.06	0.06	0.05
Annual surplus/deficit as a proportion of total revenues	0.06	0.01	-0.12	0.07	0.00	-0.05	0.05
**Faculty of Science**							

Business revenues	4,099,015	4,437,106	4,792,287	5,007,491	5,174,600	5,554,528	6,102,854
Revenues from tuition fees	338,051	382,839	369,461	517,525	337,996	330,421	415,565
Revenues from market operations	64,695	111,637	86,561	44,450	86,207	25,072	61,600
Revenues from the sale of non-financial assets	0	0	0	0	0	0	0
Revenues from financial assets and borrowing	0	0	0	0	0	0	0
Total revenues	4,099,015	4,437,106	4,792,287	5,007,491	5,174,600	5,554,528	6,102,854
Uncollected revenues	28,584	28,526	28,214	28,214	2,548	802	482
Business expenditures	4,109,869	4,362,030	4,454,648	4,435,222	4,776,993	5,263,765	5,747,864
Expenditures for business trips	114,040	113,050	103,113	23,513	45,865	139,680	180,081
Expenditure for purchase of non-financial assets	109,579	87,526	137,772	155,756	266,273	219,732	173,223
Expenditures for loan repayments	0	0	0	0	0	0	0
Total expenditure	4,219,448	4,449,556	4,592,420	4,590,978	5,043,266	5,483,498	5,921,087
Surplus of revenues available in the next period	423,452	362,070	557,768	974,281	1,089,932	1,161,524	1,342,137
Deficit of revenues to cover the next period	0	0	0	0	0	0	0
Average No. of employees	141	145	148	151	152	154	155
Share of public subsidies in total revenues	0.08	0.08	0.07	0.07	0.08	0.07	0.06
Annual surplus/deficit as a proportion of total revenues	-0.03	0.00	0.04	0.08	0.03	0.01	0.03
**Faculty of Maritime Studies**							
Business revenues	3,940,745	3,831,698	4,446,342	4,347,579	4,430,784	4,653,492	5,223,562
Revenues from tuition fees	793,549	599,376	826,147	796,015	400,030	560,559	600,704
Revenues from market operations	698,770	567,847	687,109	431,820	654,923	579,403	456,283
Revenues from the sale of non-financial assets	0	0	0	159	40	0	0
Revenues from financial assets and borrowing	0	0	0	0	2,654	0	0
Total revenues	3,940,745	3,831,698	4,446,342	4,347,738	4,433,478	4,653,492	5,223,562
Uncollected revenues	29,872	44,486	25,574	0	57,186	242,094	120,630
Business expenditures	3,584,692	4,033,215	4,209,309	3,839,610	4,362,439	4,597,153	4,954,313
Expenditures for business trips	60,735	70,570	82,356	23,290	49,788	69,679	104,979
Expenditure for purchase of non-financial assets	189,557	163,846	761,698	63,696	205,229	156,529	65,453

Expenditures for loan repayments	0	0	2,654	0	0	0	0
Total expenditure	3,774,250	4,197,062	4,973,662	3,903,306	4,567,668	4,753,682	5,019,766
Surplus of revenues available in the next period	1,301,740	863,401	263,108	780,514	646,324	546,133	749,929
Deficit of revenues to cover the next period	0	0	0	0	0	0	0
Average No. of employees	98	111	112	116	118	116	119
Share of public subsidies in total revenues	0.12	0.11	0.10	0.10	0.09	0.08	0.06
Annual surplus/deficit as a proportion of total revenues	0.04	-0.10	-0.12	0.10	-0.03	-0.02	0.04
**Faculty of Law**							
Business revenues	3,915,877	3,780,314	3,615,537	3,683,808	3,507,907	3,442,460	3,581,635
Revenues from tuition fees	1,589,384	1,474,250	1,289,844	1,218,869	1,075,708	994,209	925,900
Revenues from market operations	146,626	90,070	78,346	147,659	99,120	40,586	50,921
Revenues from the sale of non-financial assets	371	389	1,151	200	111	111	111
Revenues from financial assets and borrowing	0	0	0	0	0	0	0
Total revenues	3,916,249	3,780,703	3,616,688	3,684,008	3,508,018	3,442,571	3,581,747
Uncollected revenues	42,602	35,912	26,824	20,542	11,632	6,265	5,091
Business expenditures	3,544,074	3,496,899	3,698,303	3,487,217	3,498,528	3,415,448	3,805,446
Expenditures for business trips	82,981	61,833	57,639	19,821	29,856	57,284	60,272
Expenditure for purchase of non-financial assets	138,657	103,914	73,746	298,549	119,939	26,675	35,436
Expenditures for loan repayments	0	0	0	0	0	0	0
Total expenditure	3,682,731	3,600,813	3,772,049	3,785,766	3,618,467	3,442,123	3,840,882
Surplus of revenues available in the next period	3,651,390	3,795,616	3,596,319	3,494,641	3,300,105	3,242,703	3,244,154
Deficit of revenues to cover the next period	0	0	0	0	0	0	0
Average No. of employees	77	79	77	74	73	73	76
Share of public subsidies in total revenues	0,09	0,07	0,07	0,06	0,06	0,07	0,05
Annual surplus/deficit as a proportion of total revenues	0,06	0,05	-0,04	-0,03	-0,03	0,00	-0,07
**Art Academy**							
Business revenues	3,573,287	3,731,750	4,008,284	4,018,575	4,038,950	4,333,449	5,060,919

Revenues from tuition fees	91,181	101,526	80,883	69,460	73,601	86,419	90,619
Revenues from market operations	66,665	40,214	44,158	12,493	66,170	88,400	50,199
Revenues from the sale of non-financial assets	0	0	0	0	0	0	0
Revenues from financial assets and borrowing	0	0	0	0	0	0	0
Total revenues	3,573,287	3,731,750	4,008,284	4,018,575	4,038,950	4,333,449	5,060,919
Uncollected revenues	17,023	28,560	11,002	17,123	18,443	43,777	31,773
Business expenditures	3,308,208	3,669,627	3,862,899	4,011,984	3,958,100	4,315,351	4,793,176
Expenditures for business trips	44,851	37,215	58,160	19,600	13,580	41,570	31,676
Expenditure for purchase of non-financial assets	47,818	72,587	81,385	92,268	98,752	47,883	73,457
Expenditures for loan repayments	0	0	0	0	0	0	0
Total expenditure	3,356,025	3,742,214	3,944,284	4,104,252	4,056,852	4,363,234	4,866,633
Surplus of revenues available in the next period	91,607	81,143	144,919	53,071	35,168	6,774	192,459
Deficit of revenues to cover the next period	0	0	0	0	0	0	0
Average No. of employees	112	116	118	117	114	118	120
Share of public subsidies in total revenues	0.09	0.08	0.08	0.08	0.08	0.07	0.05
Annual surplus/deficit as a proportion of total revenues	0.06	0.00	0.02	-0.02	0.00	-0.01	0.04
**Rectorate**							
Business revenues	13,256,370	16,868,625	29,134,321	16,347,859	17,845,566	22,611,442	22,039,546
Revenues from tuition fees	1,760,344	2,527,638	2,684,102	2,906,358	2,432,582	3,748,053	3,221,827
Revenues from market operations	1,135,726	297,020	634,948	387,099	274,763	337,213	417,179
Revenues from the sale of non-financial assets	432,787	2,675,028	884,489	308	16,028	931,542	0
Revenues from financial assets and borrowing	9,954,211	0	0	0	0	0	0
Total revenues	23,643,368	19,543,653	30,018,811	16,348,166	17,861,594	23,542,984	22,039,546
Uncollected revenues	590,827	346,627	750,192	1,023,239	1,470,931	1,387,725	1,510,492
Business expenditures	8,980,252	9,801,387	11,599,555	11,603,343	12,942,585	15,503,653	18,887,622
Expenditures for business trips	140,878	208,697	242,836	90,121	173,947	426,824	407,102
Expenditure for purchase of non-financial assets	14,857,107	2,531,903	8,385,199	8,289,599	4,383,750	5,050,091	5,026,859
Expenditures for loan repayments	0	6,237,972	3,716,239	0	416,444	0	3,180

Total expenditure	23,837,359	18,571,262	23,700,993	19,892,942	17,742,779	20,553,744	23,917,660
Surplus of revenues available in the next period	0	318,389	1,926,525	0	0	1,336,311	0
Deficit of revenues to cover the next period	591,180	0	0	1,108,085	1,236,792	0	546,477
Average No. of employees	232	264	295	314	338	372	364
Share of public subsidies in total revenues	0.03	0.04	0.02	0.05	0.05	0.04	0.04
Annual surplus/deficit as a proportion of total revenues	-0.01	0.05	0.21	-0.22	0.01	0.13	-0.09

Table S4. Outgoing & Incoming mobilities of staff and students from 2016/2017 to 2022/2023 academic year

**Indicator**	**2016/2017**	**2017/2018**	**2018/2019**	**2019/2020**	**2020/2021**	**2021/2022**	**2022/2023**
**Faculty of Economics, Business, and Tourism**	**157**	**166**	**225**	**174**	**151**	**243**	**267**
Outgoing student mobility	66	67	75	81	54	82	91
Outgoing Staff mobility	25	32	44	20	18	63	61
Incoming student mobility	65	60	70	73	77	95	112
Incoming staff mobility	1	7	36	0	2	3	3
**Faculty of Electrical Engineering, Mechanical Engineering and Naval Architecture**	**55**	**52**	**86**	**60**	**50**	**79**	**97**
Outgoing student mobility	27	20	43	27	26	27	48
Outgoing Staff mobility	12	11	11	5	1	14	21
Incoming student mobility	16	13	29	28	14	36	26
Incoming staff mobility	0	8	3	0	9	2	2
**Faculty of Civil Engineering, Architecture and Geodesy**	**53**	**39**	**52**	**35**	**24**	**50**	**40**
Outgoing student mobility	16	25	17	14	15	16	21
Outgoing Staff mobility	10	9	12	15	2	17	7
Incoming student mobility	23	5	13	6	4	14	12
Incoming staff mobility	4	0	10	0	3	3	0
**Faculty of Humanities and Social Sciences**	**105**	**74**	**122**	**69**	**50**	**91**	**89**
Outgoing student mobility	59	45	29	36	25	21	19
Outgoing Staff mobility	12	15	34	16	6	21	14
Incoming student mobility	24	12	20	16	19	42	49
Incoming staff mobility	10	2	39	1	0	7	7
**Catholic Faculty of Theology**	**9**	**7**	**6**	**4**	**8**	**26**	**25**
Outgoing student mobility	4	5	5	3	5	2	7
Outgoing Staff mobility	1	0	1	1	1	22	12
Incoming student mobility	3	0	0	0	0	1	1
Incoming staff mobility	1	2	0	0	2	1	5

**Faculty of Chemistry and Technology**	**3**	**14**	**14**	**9**	**22**	**30**	**28**
Outgoing student mobility	1	5	6	1	17	17	13
Outgoing Staff mobility	0	4	2	4	2	7	10
Incoming student mobility	0	4	2	0	1	5	5
Incoming staff mobility	2	1	4	4	2	1	0
**Faculty of Kinesiology**	**36**	**51**	**77**	**31**	**25**	**44**	**63**
Outgoing student mobility	11	19	14	9	7	11	9
Outgoing Staff mobility	15	14	29	7	0	7	11
Incoming student mobility	9	13	15	14	17	26	37
Incoming staff mobility	1	5	19	1	1	0	6
**University of Split, School of Medicine**	**13**	**31**	**57**	**18**	**35**	**59**	**61**
Outgoing student mobility	3	7	32	3	17	13	16
Outgoing Staff mobility	5	14	12	8	6	24	25
Incoming student mobility	3	9	9	7	9	12	17
Incoming staff mobility	2	1	4	0	3	10	3
**Faculty of Maritime Studies**	**50**	**58**	**82**	**56**	**46**	**70**	**70**
Outgoing student mobility	7	10	10	4	4	20	16
Outgoing Staff mobility	12	17	22	25	6	11	22
Incoming student mobility	28	29	33	27	33	39	27
Incoming staff mobility	3	2	17	0	3	0	5
**Faculty of Law**	**13**	**23**	**30**	**18**	**17**	**58**	**32**
Outgoing student mobility	6	10	15	15	4	13	8
Outgoing Staff mobility	0	0	1	0	0	2	4
Incoming student mobility	6	13	12	3	13	22	19
Incoming staff mobility	1	0	2	0	0	21	1
**Faculty of Science**	**13**	**13**	**18**	**13**	**18**	**27**	**41**
Outgoing student mobility	6	3	6	5	10	16	18
Outgoing Staff mobility	2	4	3	4	1	2	7
Incoming student mobility	5	6	3	4	5	9	16
Incoming staff mobility	0	0	6	0	2	0	0
**University Department of Professional Studies**	**47**	**59**	**119**	**40**	**34**	**105**	**133**
Outgoing student mobility	15	9	15	5	9	4	16
Outgoing Staff mobility	11	20	23	22	13	52	46
Incoming student mobility	15	30	55	13	7	39	59
Incoming staff mobility	6	0	26	0	5	10	12
**University Department of Marine Studies**	**0**	**8**	**16**	**1**	**1**	**17**	**13**
Outgoing student mobility	0	5	7	0	0	1	3
Outgoing Staff mobility	0	3	5	1	0	5	4
Incoming student mobility	0	0	2	0	1	10	6
Incoming staff mobility	0	0	2	0	0	1	0
**University Department of Health Studies**	**9**	**8**	**11**	**6**	**4**	**24**	**29**
Outgoing student mobility	3	0	1	0	0	0	1
Outgoing Staff mobility	0	0	0	1	1	10	15
Incoming student mobility	6	7	8	5	3	10	13
Incoming staff mobility	0	1	2	0	0	4	0

**University Department for Forensic Sciences**	**1**	**1**	**1**	**0**	**0**	**1**	**12**
Outgoing student mobility	1	1	0	0	0	0	2
Outgoing Staff mobility	0	0	1	0	0	0	3
Incoming student mobility	0	0	0	0	0	1	2
Incoming staff mobility	0	0	0	0	0	0	5
**Art Academy**	**30**	**23**	**48**	**27**	**18**	**27**	**58**
Outgoing student mobility	10	8	10	9	3	6	29
Outgoing Staff mobility	8	9	12	7	4	7	13
Incoming student mobility	9	4	7	11	10	11	16
Incoming staff mobility	1	2	19	0	1	3	0
**Rectorate (Administrative unit)**	**75**	**80**	**90**	**61**	**51**	**184**	**127**
Outgoing student mobility	0	0	0	0	0	0	0
Outgoing Staff mobility	12	16	10	24	10	41	13
Incoming student mobility	1	0	0	0	0	0	0
Incoming staff mobility	62	64	80	37	41	143	114

Abbreviations: STEM – science, technology, engineering, and mathematics + biomedicine and healthcare; SH – social sciences and humanities; ART – art

Figure S1. The results of the interrupted time series analysis*

Variable	Interrupted time series analysis	Data for individual university constituents
**Academic indicators (data for academic years 2016/2017 to 2022/2023)**		
No. students, first enrollment	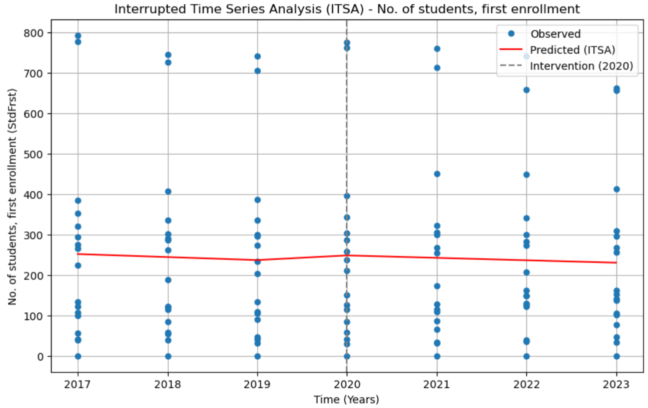	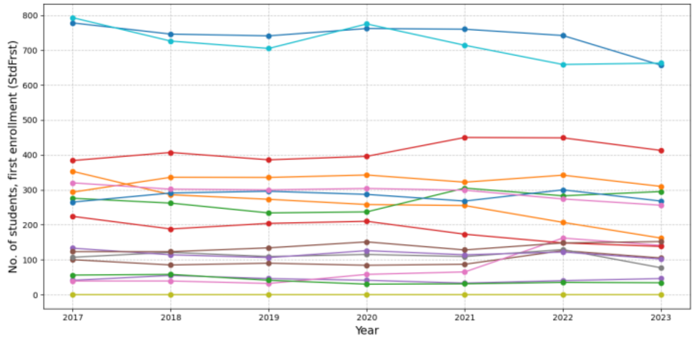 P=0.474, Friedman test
No. students, senior years	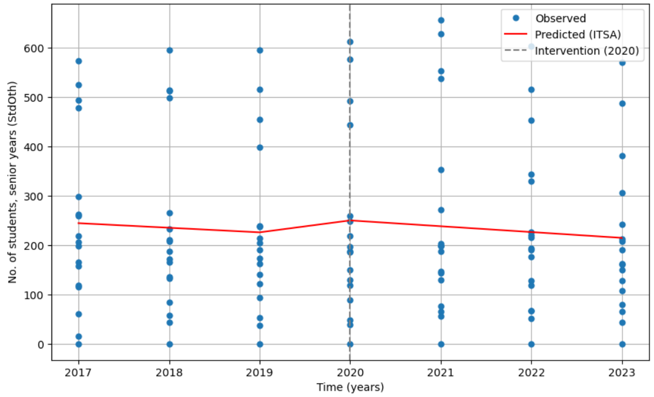	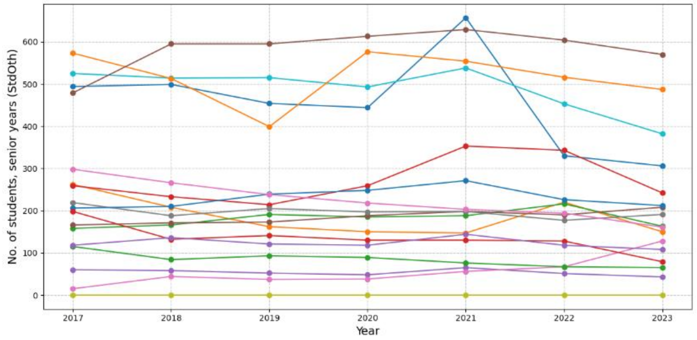 P=0.014, Friedman test; Conover post-hoc comparison (P≤0.05): years 2017-2021 vs 2023

Total No. of students receiving public subsidy	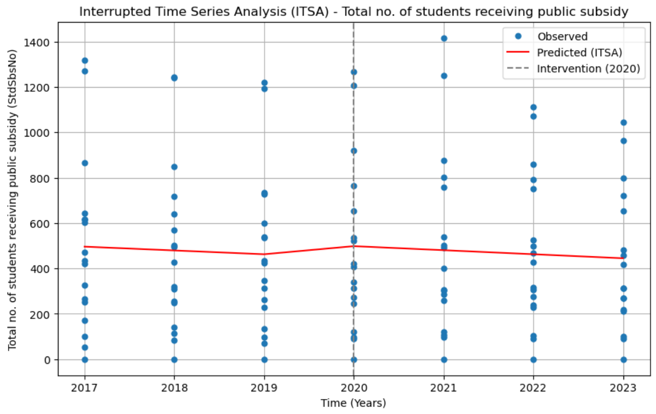	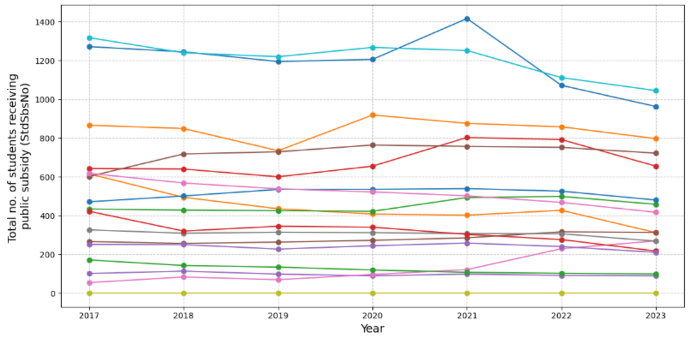<br>P=0.017, Friedman test. Conover post-hoc comparison (P≤0.05): years 2017, 2018, 2020, 2021 vs 2023
Total public subsidy amount (Euro × 10^7)	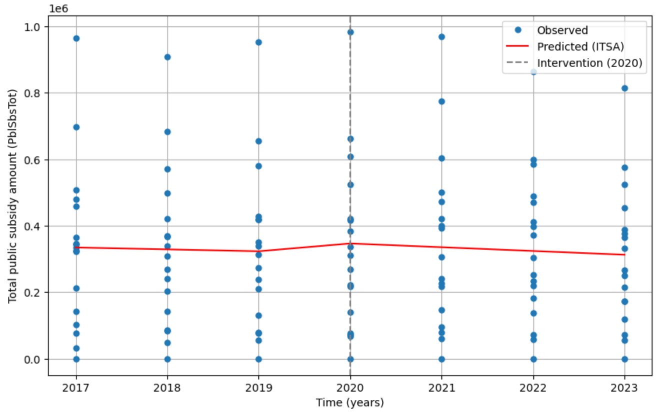	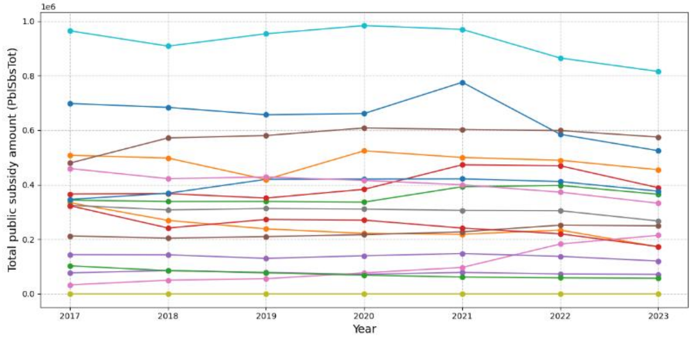<br>P=0.037, Friedman test; Conover post-hoc comparison (P≤0.05): years 2017, 2020, 2021 vs 2023

<table>
<tr>
<td>Total No. students enrolled</td>
<td>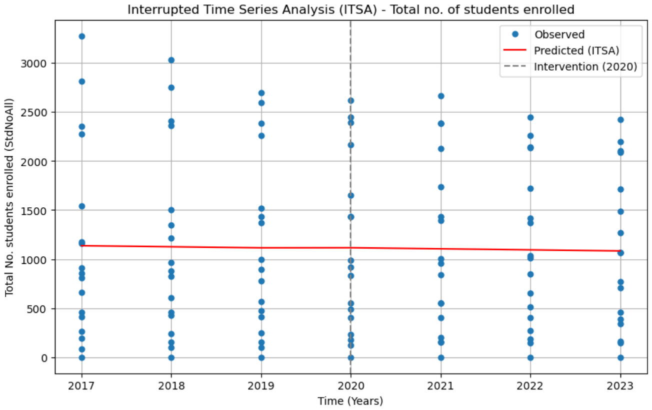
</td>
<td>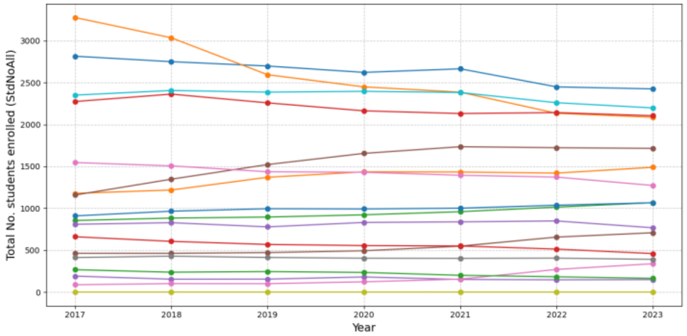

P=0.766, Friedman test</td>
</tr>
<tr>
<td>No. of students who gave up studies in the first study year</td>
<td>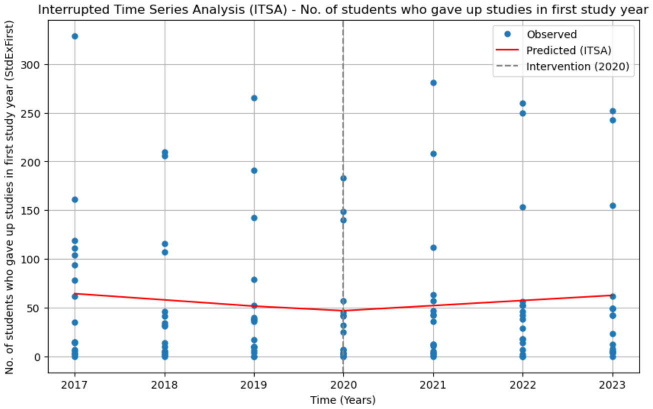
</td>
<td>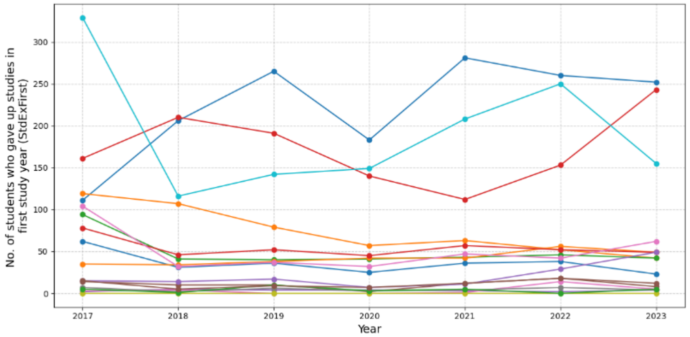

P≤0.001, Friedman test; Conover post-hoc comparison (P≤0.05): 2017 vs years 2018-2020; 2018 vs 2022; 2020 vs 2021-2023</td>
</tr>
</table>

No. students who gave up studies in any study year	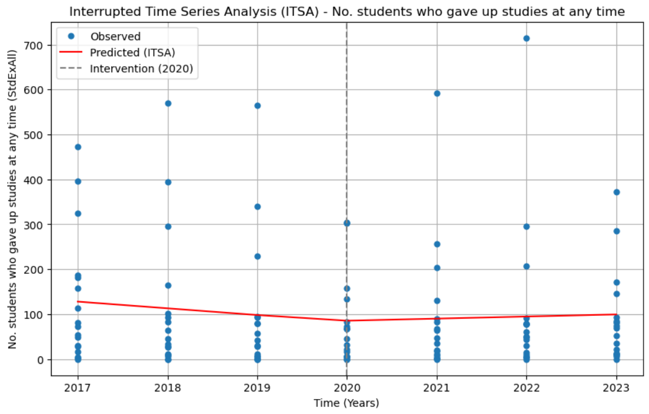	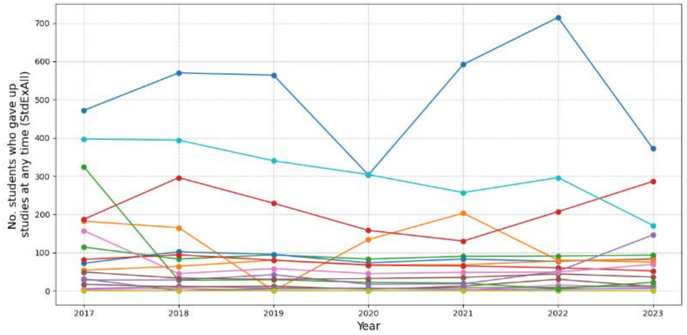<br>P=0.030, Friedman test; Conover post-hoc comparison (P≤0.05): 2017 vs years 2020-2021; 2018 vs 2021; 2020 vs 2022-2023
No. students who did not fulfill requirements for higher study year (<42 ECTS points)	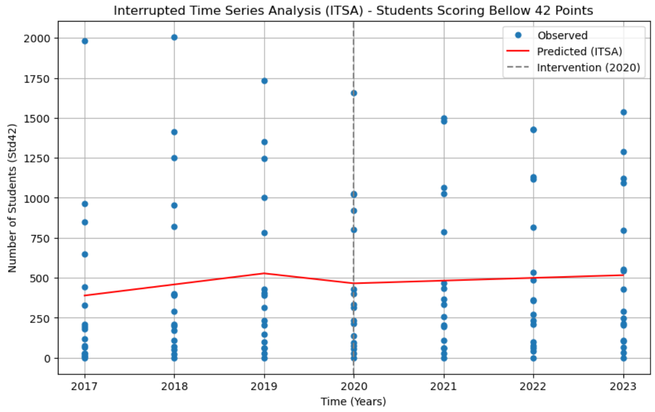	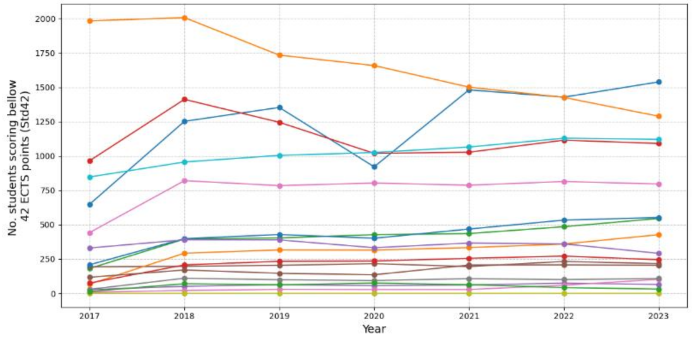<br>P<0.001, Friedman test; Conover post-hoc comparison (P≤0.05): 2017 vs years 2018-2020; 2018 vs 2022; 2019 vs 2020; 2020 vs 2021-2023

No. students who fulfilled requirements for higher study year (>60 ECTS points)	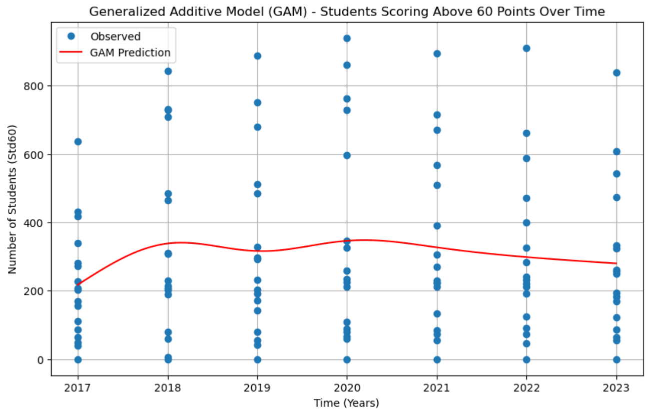	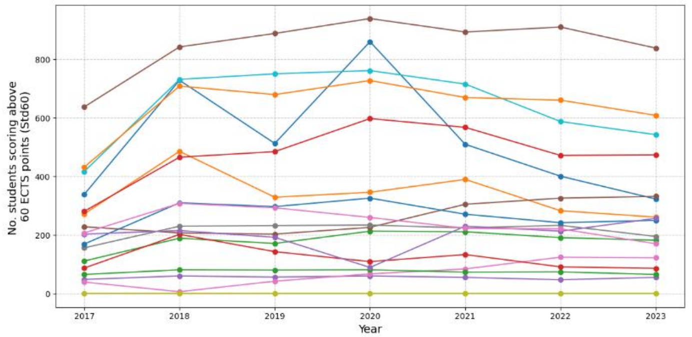<br>P<0.001, Friedman test; Conover post-hoc comparison (P≤0.05): 2017 vs years 2018-2022; 2018 vs 2023; 2019 vs 2020, 2023; 2020 vs 2022-2023; 2021 vs 2023
No. of students who graduated	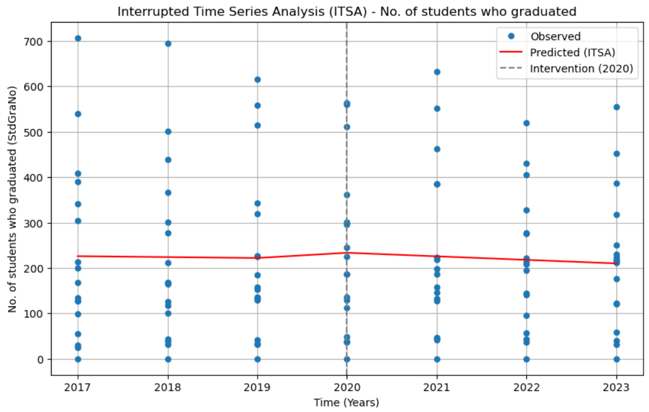	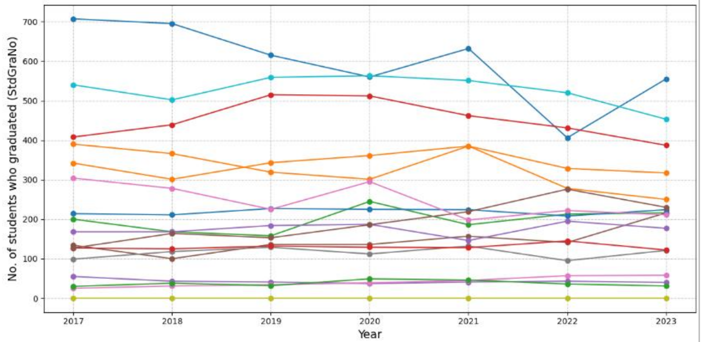<br>P=0.218, Friedman test
**Research indicators (data for calendar years 2017 to 2022)**		

Total No. academic staff	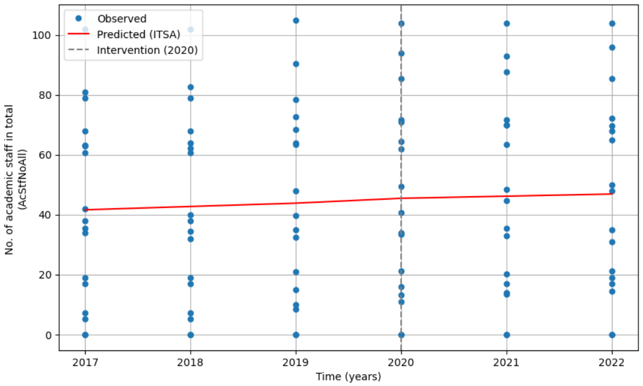	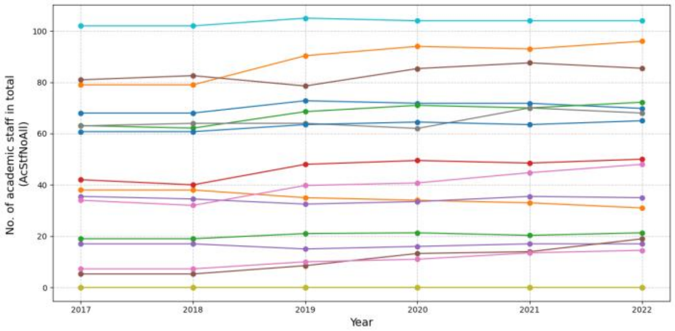 P<0.001, Friedman test. Conover post-hoc comparison (P≤0.05): years 2017, 2018, 2019 vs 2022
No. of academic staff (STEM+health)	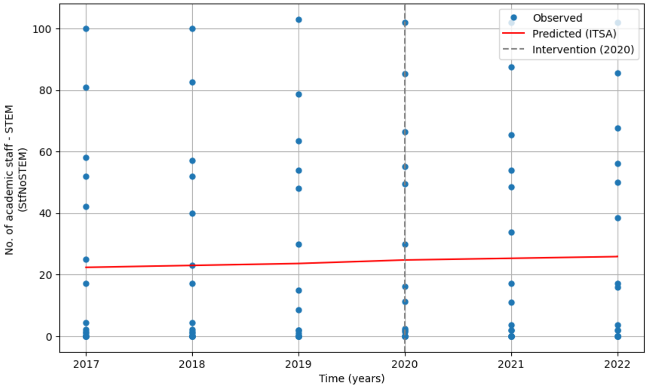	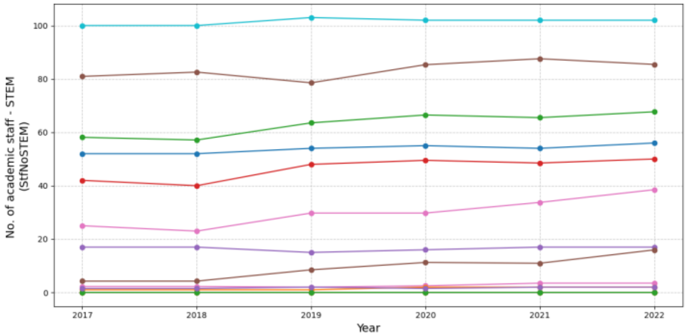 P<0.001, Friedman test; Conover post-hoc comparison (P≤0.05): 2017 vs years 2020-2022; 2018 vs 2022; 2019 vs 2021-2022

No. of academic staff (SH)	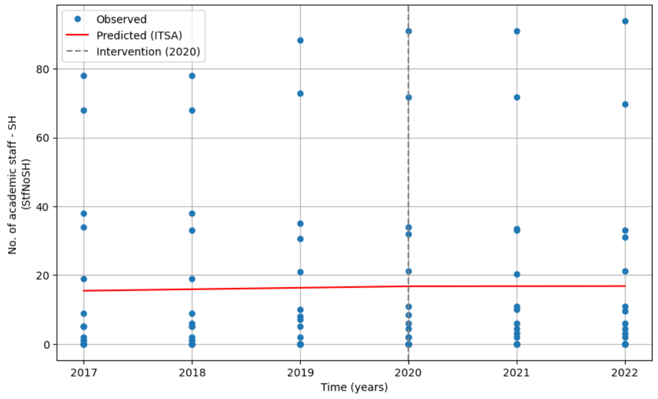	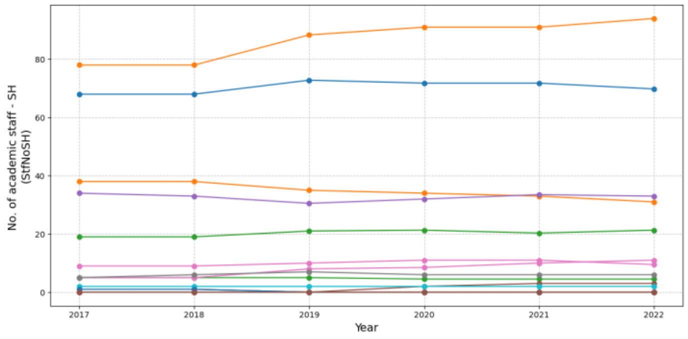<br>P=0.726, Friedman test
No. of academic staff (ART)	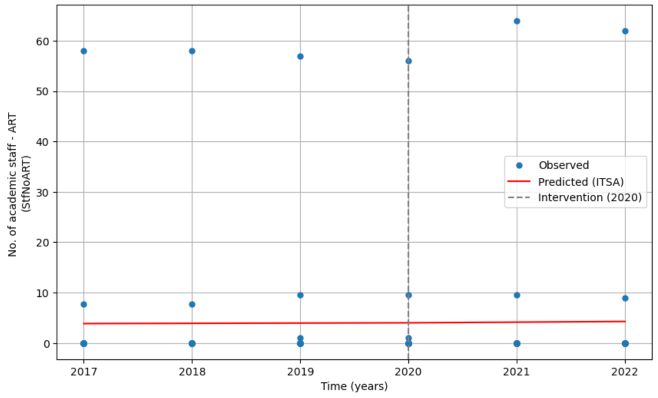	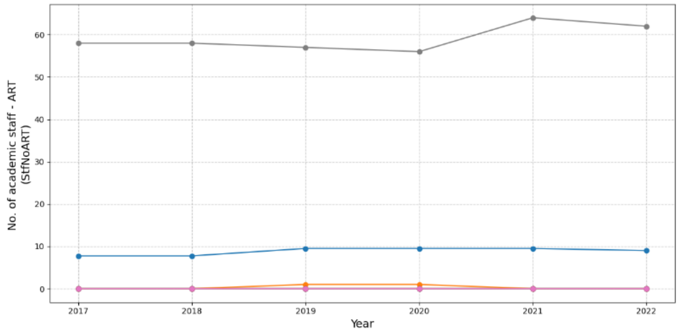<br>P=0.603, Friedman test

No. of publications in STEM+health journals	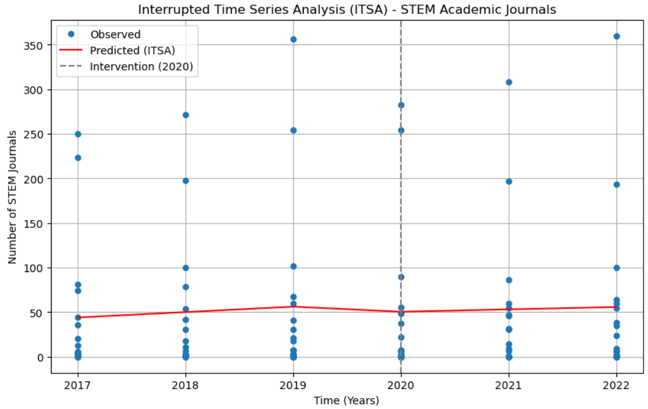	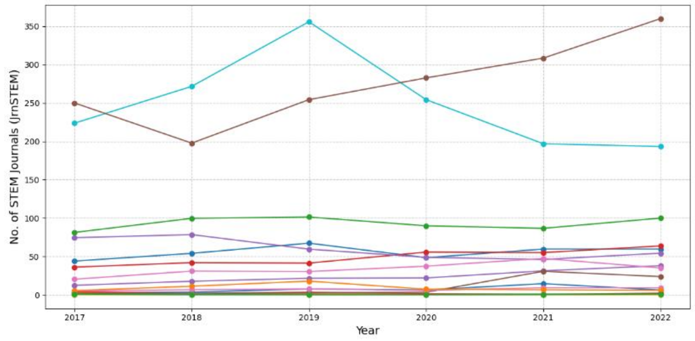<br>P=0.093, Friedman test
No. of publications in SH journals	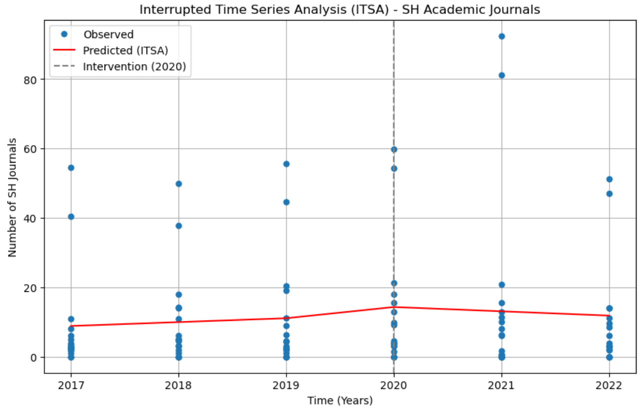	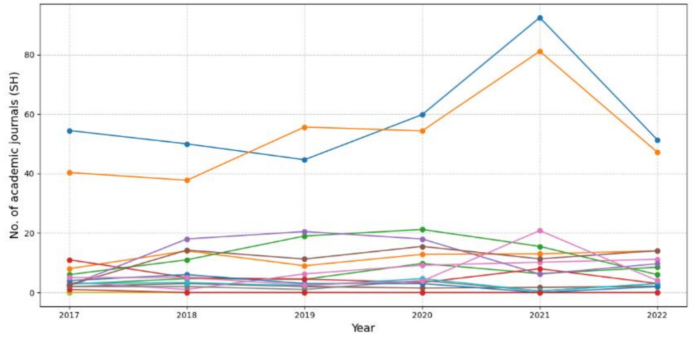<br>P=0.455, Friedman test

No. of publications in ART academic journals

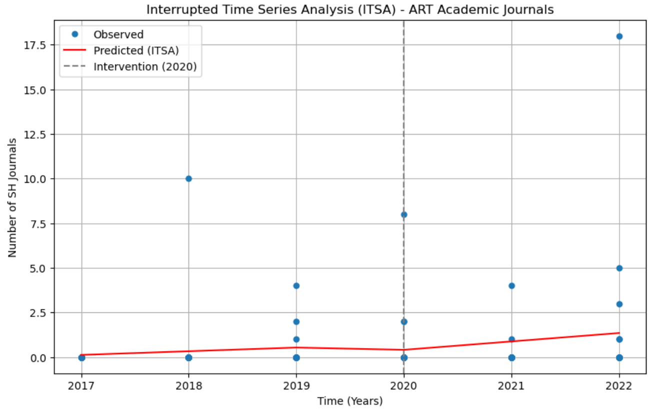

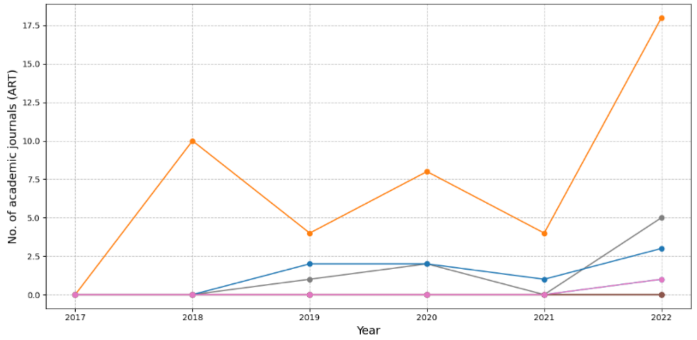

P=0.003, Friedman test; Conover post-hoc comparison (P≤0.05): 2017 vs year 2022; 2018 vs 2022; 2019 vs 2022; 2020 vs 2022; 2021 vs 2022

Legend (refers to all indicators except Financial):

- Faculty of Economics, Business, and Tourism
- Faculty of Electrical Engineering, Mechanical Engineering and Naval Architecture
- Faculty of Civil Engineering, Architecture and Geodesy
- Faculty of Humanities and Social Sciences
- Catholic Faculty of Theology
- Faculty of Chemistry and Technology
- Faculty of Kinesiology
- University of Split, School of Medicine
- Faculty of Maritime Studies
- Faculty of Law
- Faculty of Science
- University Department of Professional Studies
- University Department of Marine Studies
- University Department of Health Studies
- University Department for Forensic Sciences
- Art Academy
- Rectorate

Financial indicators (data for business years 2017 to 2023)‡

Total revenues	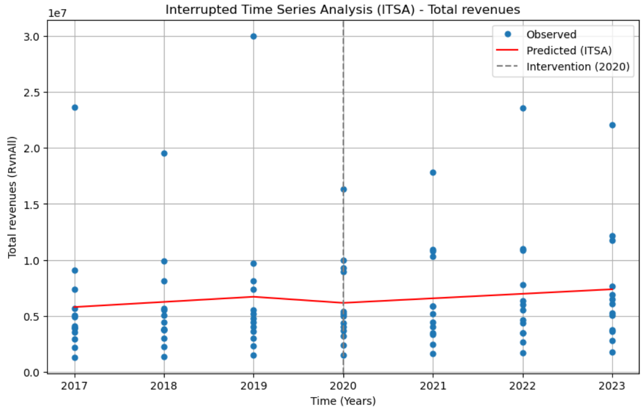	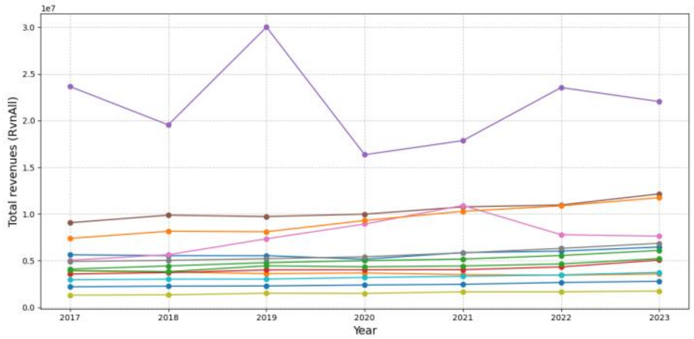<br>P<0.001, Friedman test; Conover post-hoc comparison (P≤0.05): 2017 vs years 2020-2023; 2018 vs 2021-2023; 2019 vs 2022-2023; 2020 vs 2022-2023; 2021 vs 2023
Business revenues	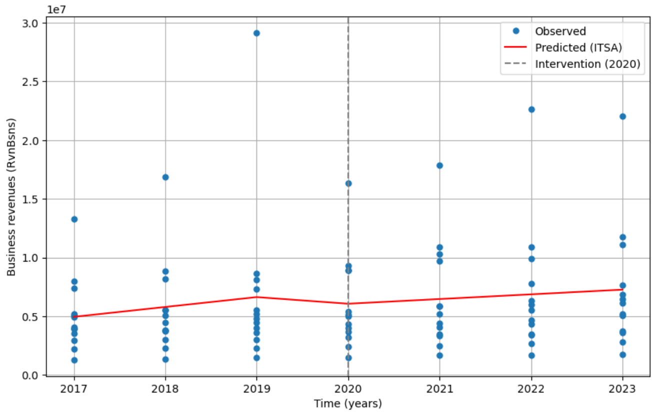	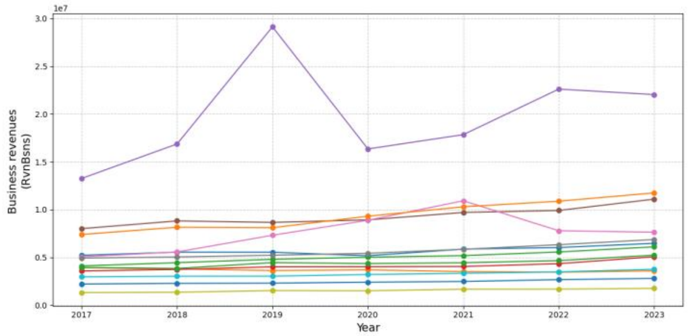<br>P<0.001, Friedman test; Conover post-hoc comparison (P≤0.05): 2017 vs years 2019-2023; 2018 vs 2021-2023; 2019 vs 2022-2023; 2020 vs 2021-2023; 2021 vs 2023

<table>
<tr>
<td>Revenues from tuition fees</td>
<td>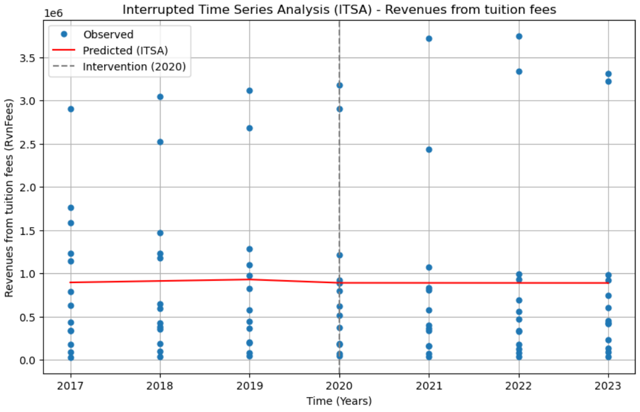
</td>
<td>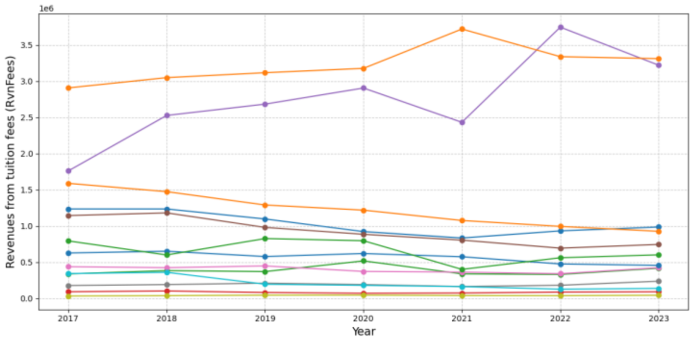

P=0.012, Friedman test; Conover post-hoc comparison (P≤0.05): 2017 vs years 2021-2022; 2018 vs 2021-2022; 2019 vs 2021-2022; 2020 vs 2021-2022</td>
</tr>
<tr>
<td>Revenues from market operations</td>
<td>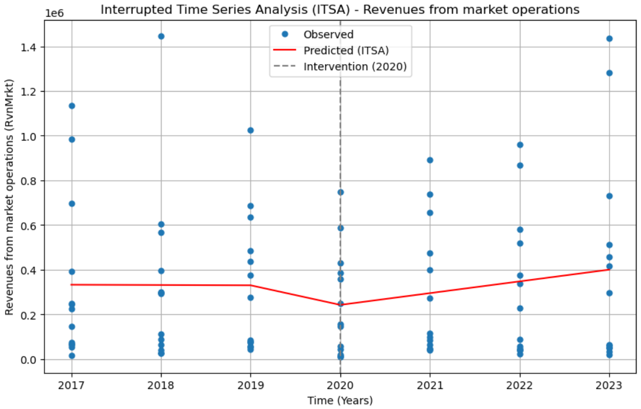
</td>
<td>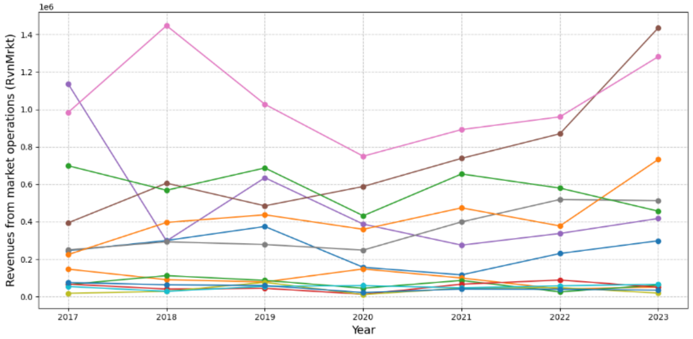

P=0.163, Friedman test</td>
</tr>
</table>

<table>
<tr>
<td>Revenues from the sale of non-financial assets</td>
<td>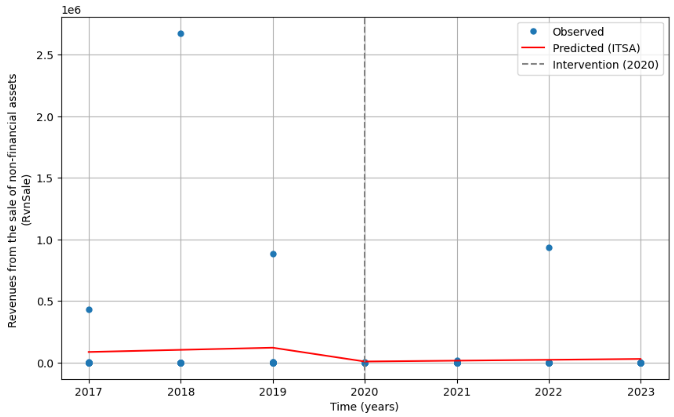
</td>
<td>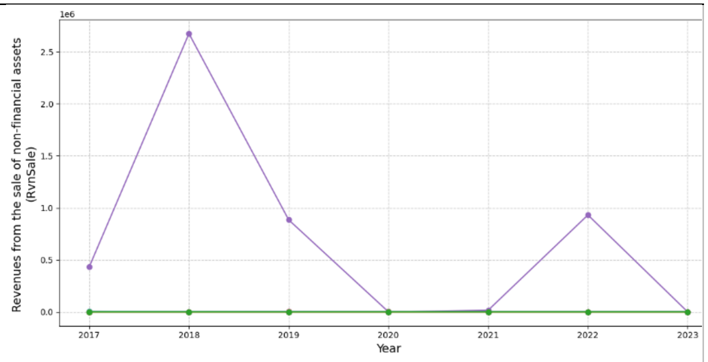

P=0.048, Friedman test</td>
</tr>
<tr>
<td>Uncollected revenues</td>
<td>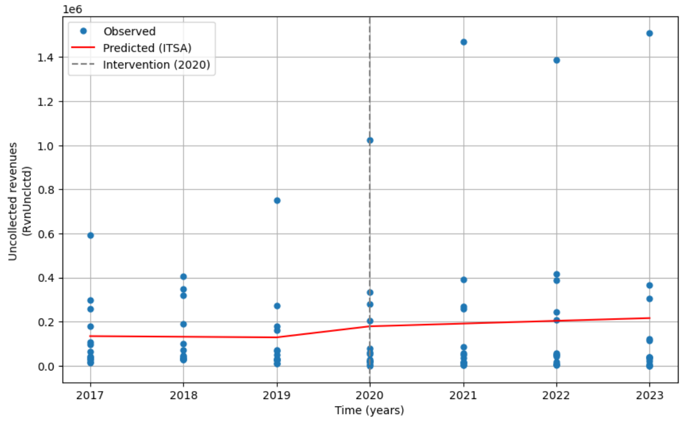
</td>
<td>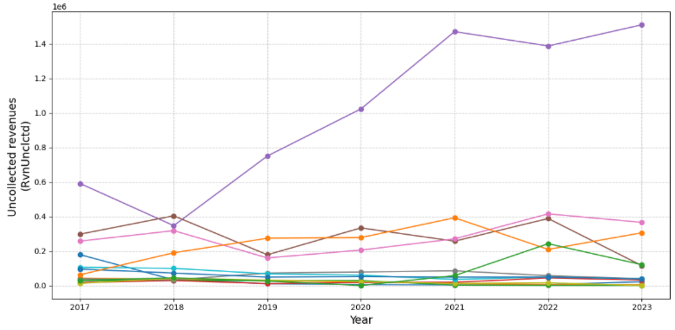

P=0.073, Friedman test</td>
</tr>
</table>

<table>
<tr>
<td>Total expenditure</td>
<td>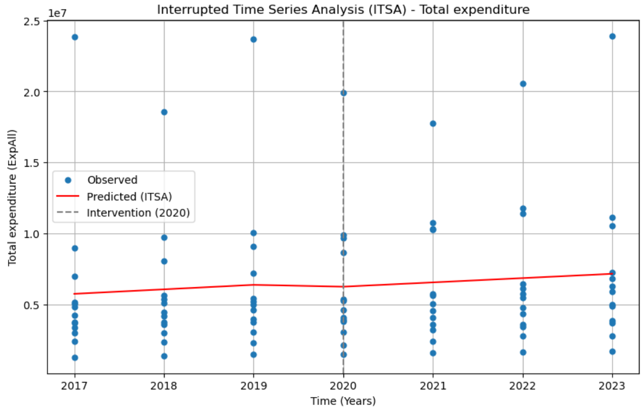
</td>
<td>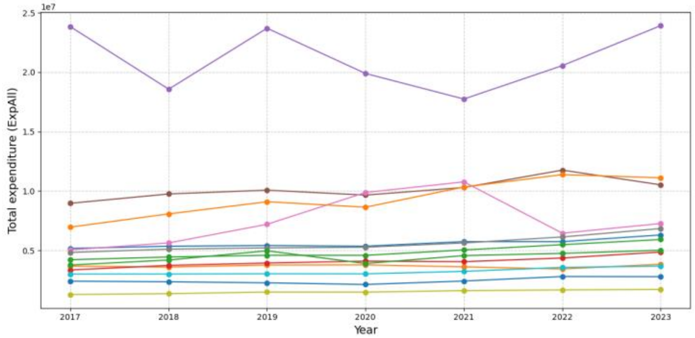

P<0.001, Friedman test; Conover post-hoc comparison (P≤0.05): 2017 vs years 2019-2023; 2018 vs 2019-2023</td>
</tr>
<tr>
<td>Business expenditures</td>
<td>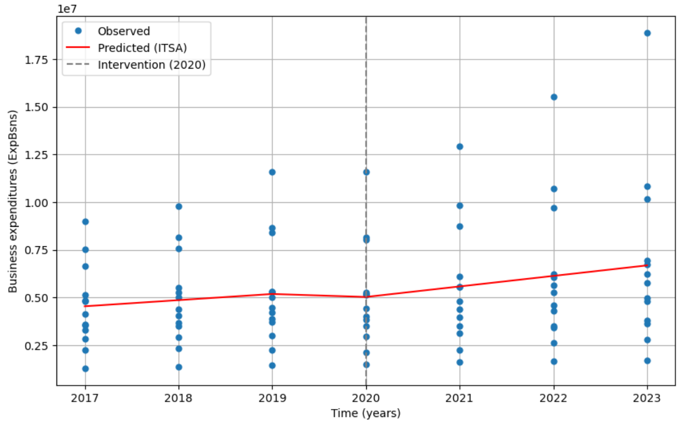
</td>
<td>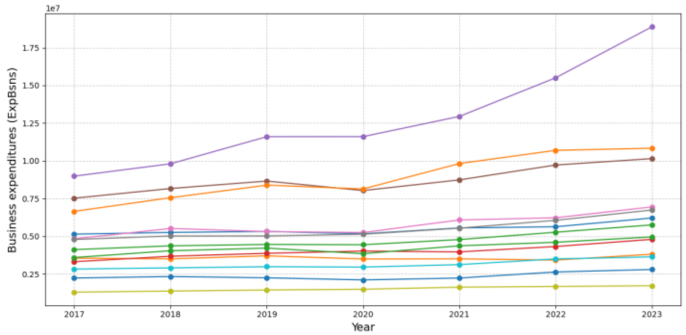

P<0.001, Friedman test; Conover post-hoc comparison (P≤0.05): 2017 vs all other years; 2018 vs 2019; 2021-2023; 2019 vs 2022-2023; 2020 vs 2021-2023; 2021 vs years 2022-2023; 2022 vs 2023</td>
</tr>
</table>

<table>
<tr>
<td>Expenditures for business trips</td>
<td>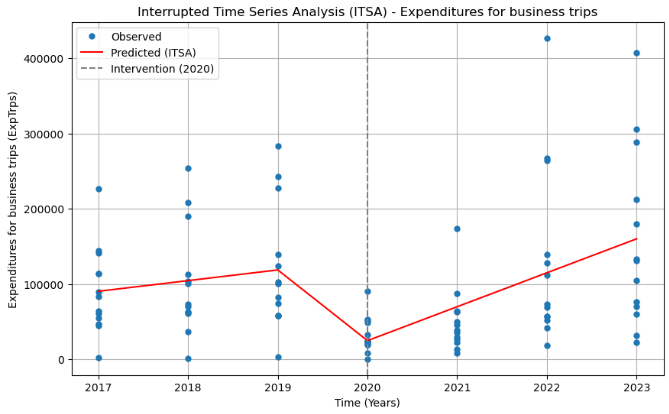
</td>
<td>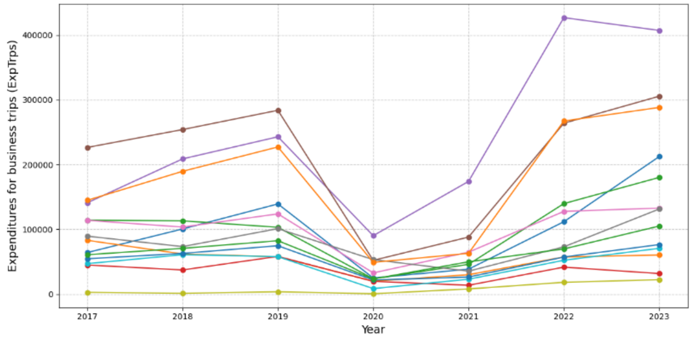

P<0.001, Friedman test; Conover post-hoc comparison (P≤0.05): 2017 vs years 2019, 2020-2023; 2018 vs 2019-2021, 2023; 2020 vs 2022-2023; 2021 vs 2022-2023; 2022 vs 2023</td>
</tr>
<tr>
<td>Expenditures for purchase of non-financial assets</td>
<td>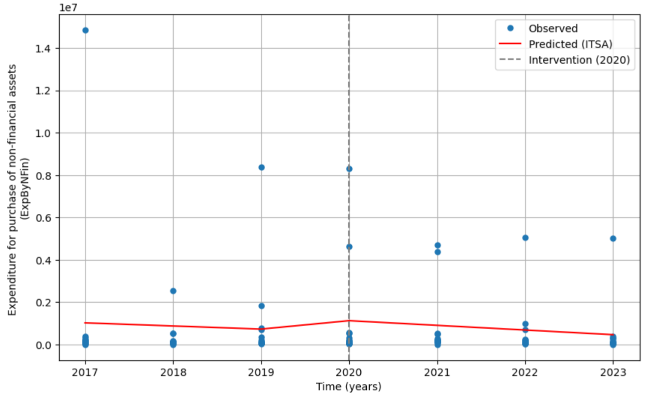
</td>
<td>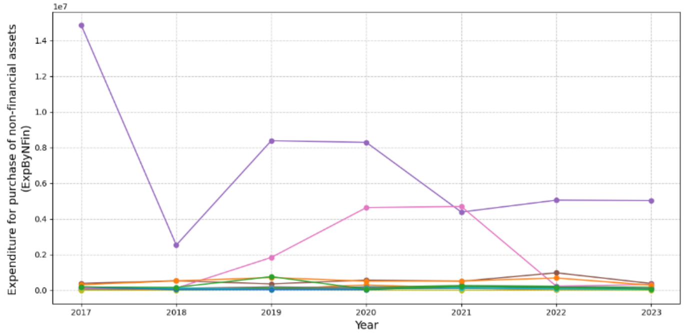

P=0.107, Friedman test</td>
</tr>
</table>

Surplus of revenues available in the next period	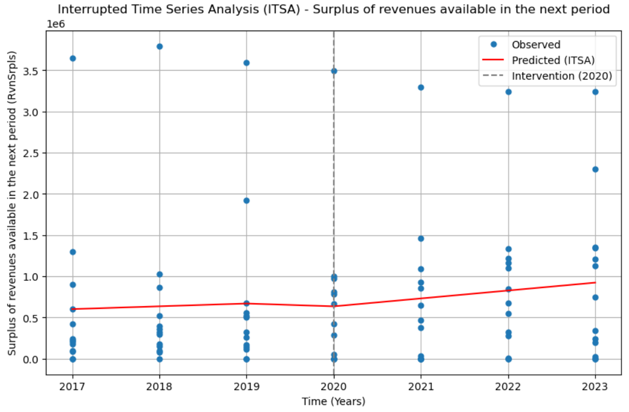	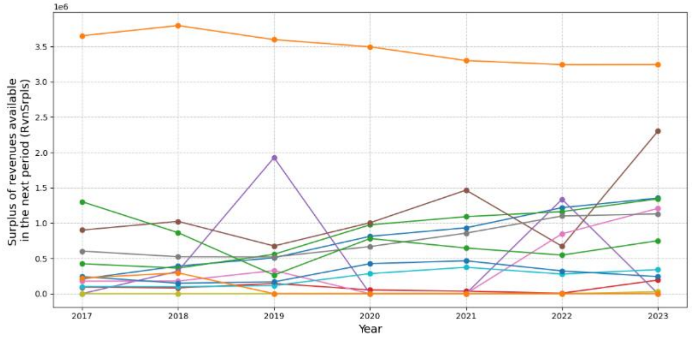 P=0.296, Friedman test
Deficit of revenues to cover the next period	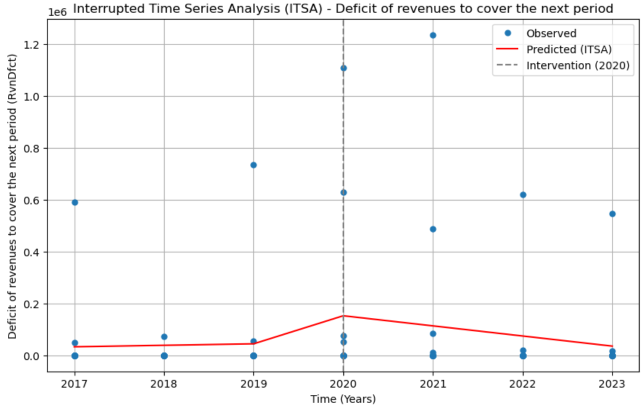	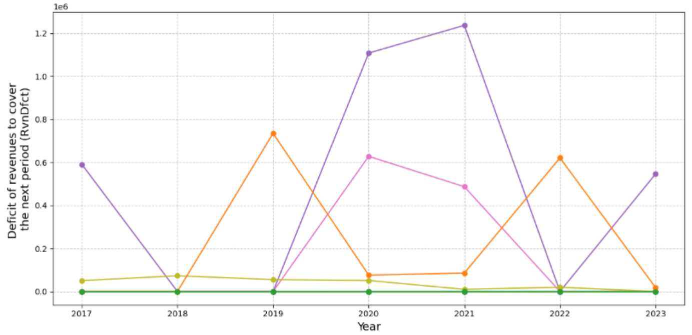 P=0.437, Friedman test

Average No. of employees	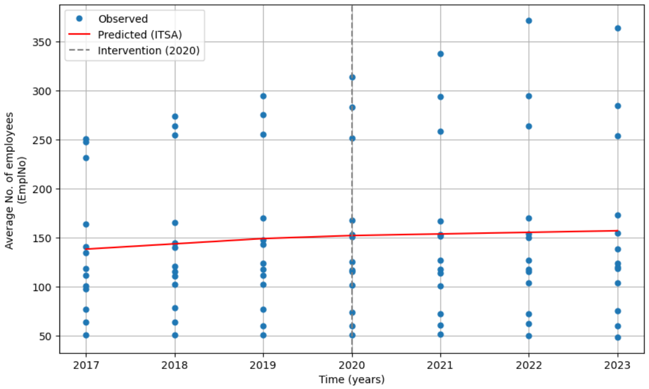	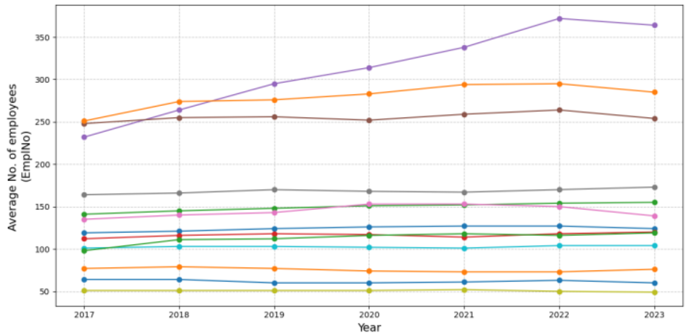<br>P=0.004, Friedman test; Conover post-hoc comparison (P≤0.05): 2017 vs years 2019-2023
Share of public subsidies in total revenues	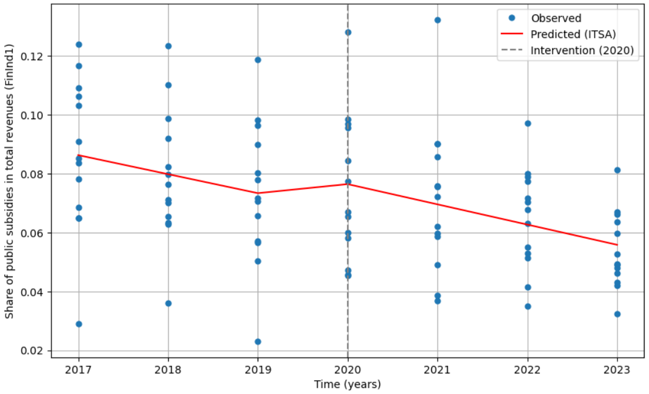	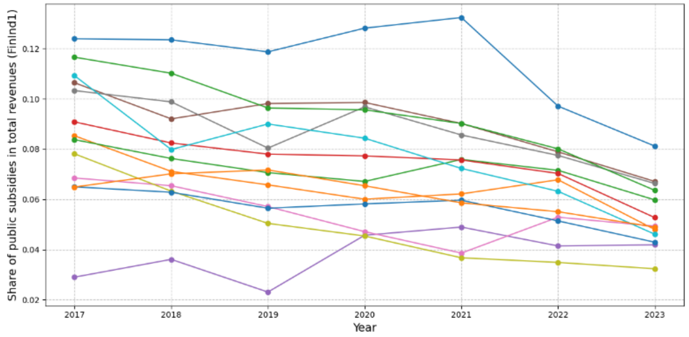<br>P<0.001, Friedman test; Conover post-hoc comparions (P≤0.05): 2023 vs years 2017-2021; 2022 vs 2017,2018; 2017 vs. 2021-2023.

Annual surplus/deficit as a proportion of total revenues	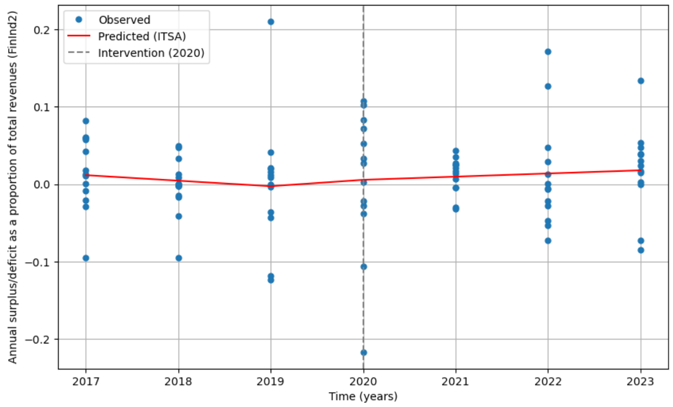	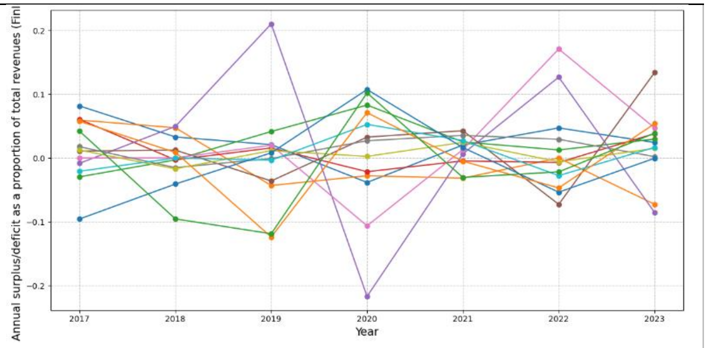 P=0.7049, Friedman test

Legend for financial indicators:

- Faculty of Economics, Business, and Tourism
- Faculty of Electrical Engineering, Mechanical Engineering and Naval Architecture
- Faculty of Civil Engineering, Architecture and Geodesy
- Faculty of Humanities and Social Sciences
- Catholic Faculty of Theology
- Faculty of Chemistry and Technology
- Faculty of Kinesiology
- University of Split, School of Medicine
- Faculty of Maritime Studies
- Faculty of Law
- Faculty of Science
- Art Academy
- Rectorate

Mobility indicators (data for academic years 2016/2017 to 2022/2023)

<table>
<tr>
<td>No. outgoing student mobilities</td>
<td>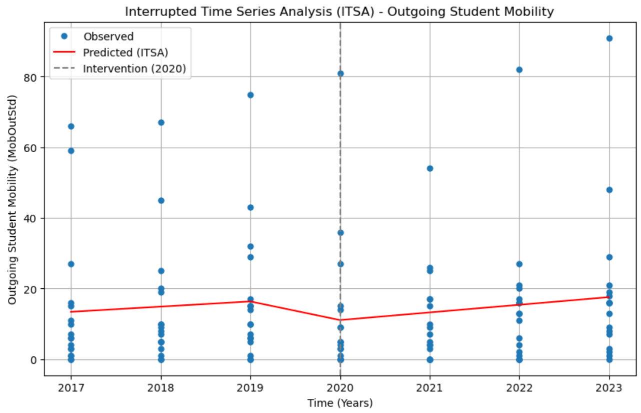
</td>
<td>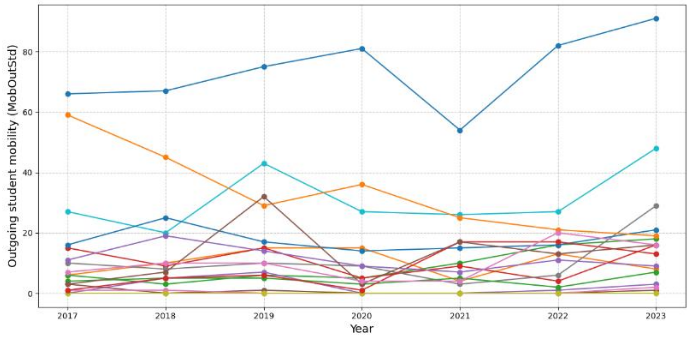

P=0.002, Friedman test; Conover post-hoc comparison (P≤0.05): 2018 vs 2023; 2019 vs 2020-2021; 2020 vs 2023; 2021 vs 2023; 2022 vs 2023</td>
</tr>
<tr>
<td>No. outgoing staff mobilities</td>
<td>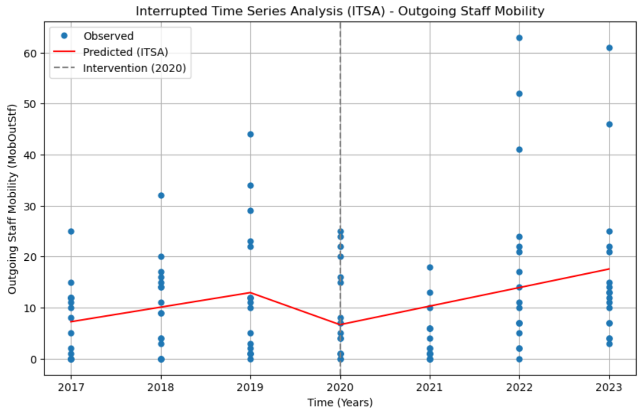
</td>
<td>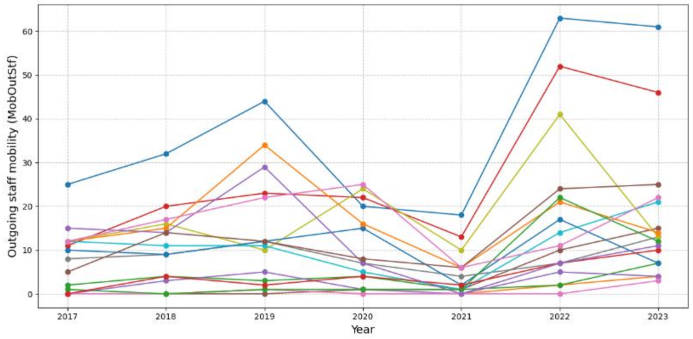

P<0.001, Friedman test; Conover post-hoc comparison (P≤0.05): 2019 vs years 2021-2023; 2019 vs. 2021, 2023; 2020 vs years 2021-2023; 2021 vs years 2022-2023</td>
</tr>
</table>

<table>
<tr>
<td>No. incoming student mobilities</td>
<td>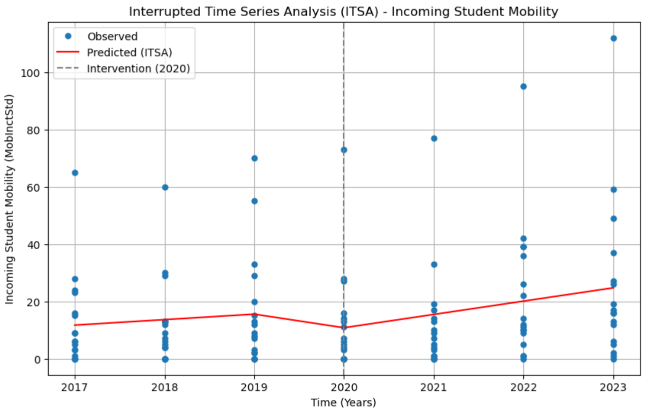
</td>
<td>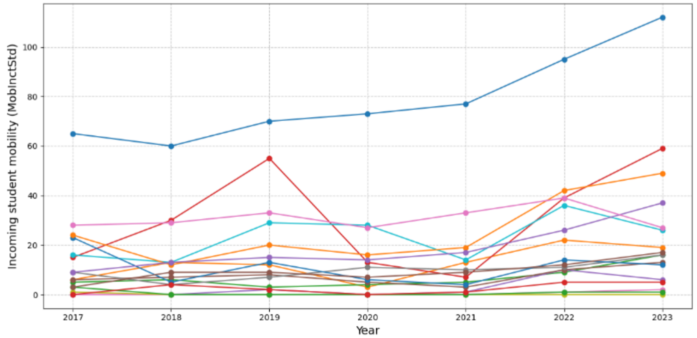

P<0.001, Friedman test; Conover post-hoc comparison (P≤0.05): 2018 vs years 2019, 2022-2023; 2019 vs years 2020, 2022-2023; 2020 vs years 2022-2023; 2021 vs years 2022-2023</td>
</tr>
<tr>
<td>No. incoming staff mobility</td>
<td>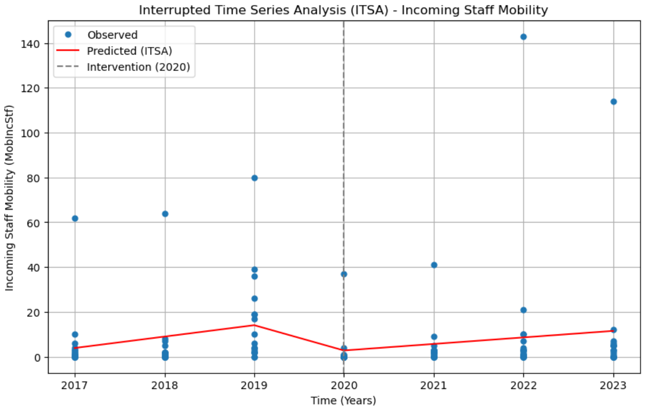
</td>
<td>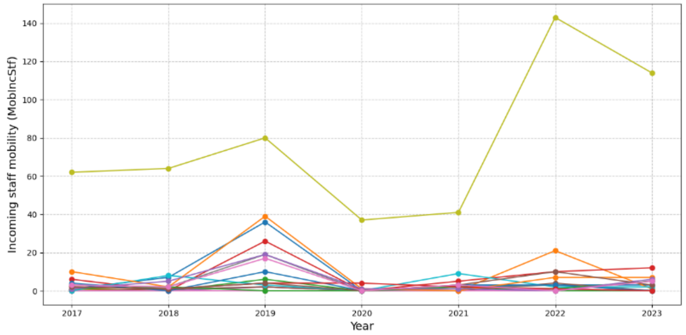

P<0.001, Friedman test; Conover post-hoc comparison (P≤0.05): 2018 vs years 2019-2020; 2019 vs years 2020-2023; 2020 vs years 2021-2023</td>
</tr>
</table>

*Interrupted Time Series Analysis (ITSA) was applied to assess trends in student success, research productivity, finances, and mobility at the University of Split before, during, and after the pandemic. Data on individual constituent levels are graphically presented for each observed indicator. To detect differences in medians for individual indicators over years, the Friedman test and Conover post-hoc test were applied, and results are disclosed below each graph.

†Two indicators (Revenues from financial assets and borrowing and Expenditures for loan repayments) in Supplementary Table 3 were not subject to analyses due to the small amount of data.

‡Financial indicators were analyzed for 13 constituents of the University of Split. Four departments are not obligated to draft official financial reports, but their activities are monitored and recorded within the Rectorate´s business books.

Figure S2. Academic indicators for the University of Split from 2016/2017 to 2022/2023 academic year. Blue vertical line indicates the start of the COVID-19 pandemic.

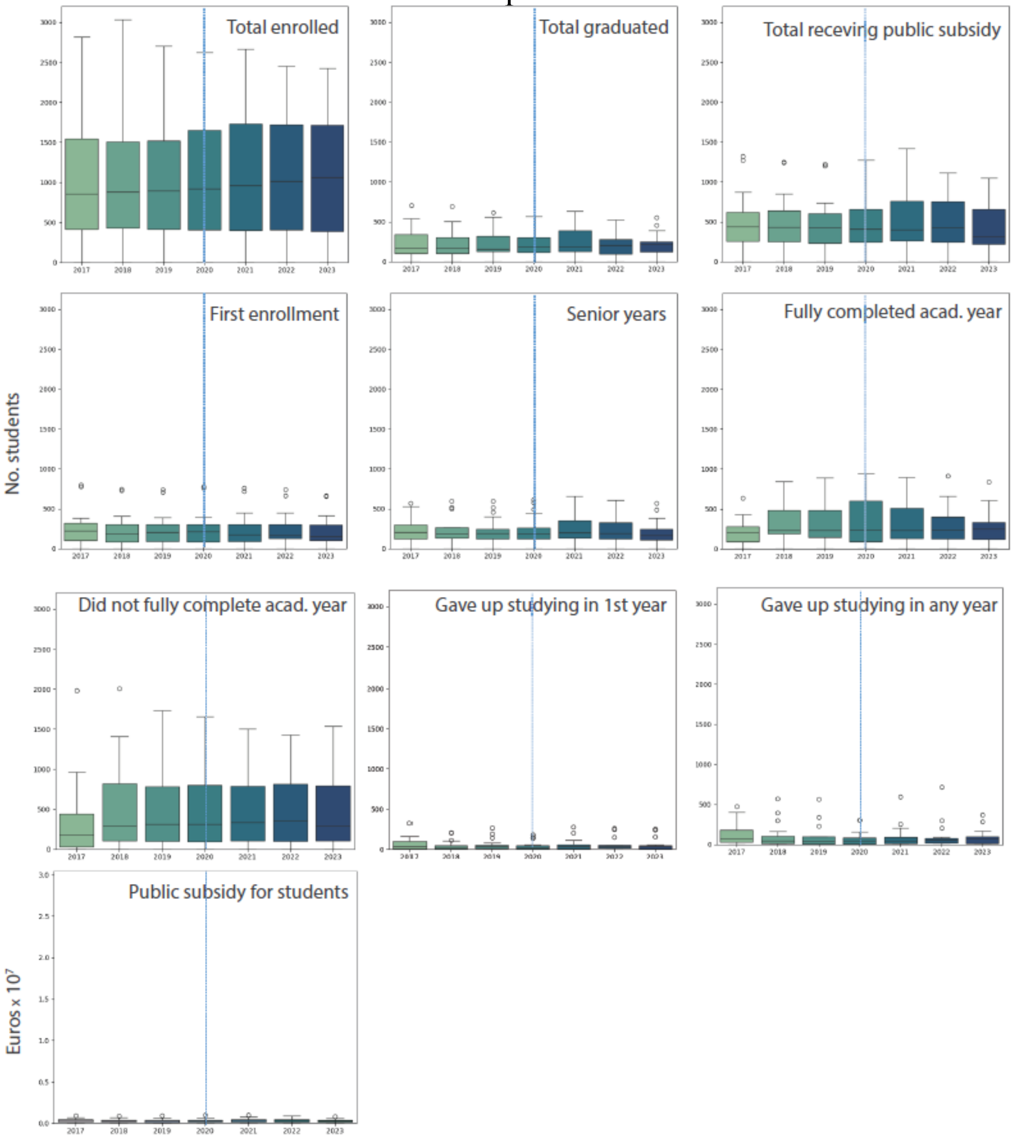

Figure S3. Academic staff and scientific output of the University of Split from 2017 to 2022 calendar year. These indicators were calculated for academic years until 2022 because only data for these years were available at the university level. The data on journal articles are from the Web of Science Core Collection and Scopus. Blue vertical line indicates the start of the COVID-19 pandemic.

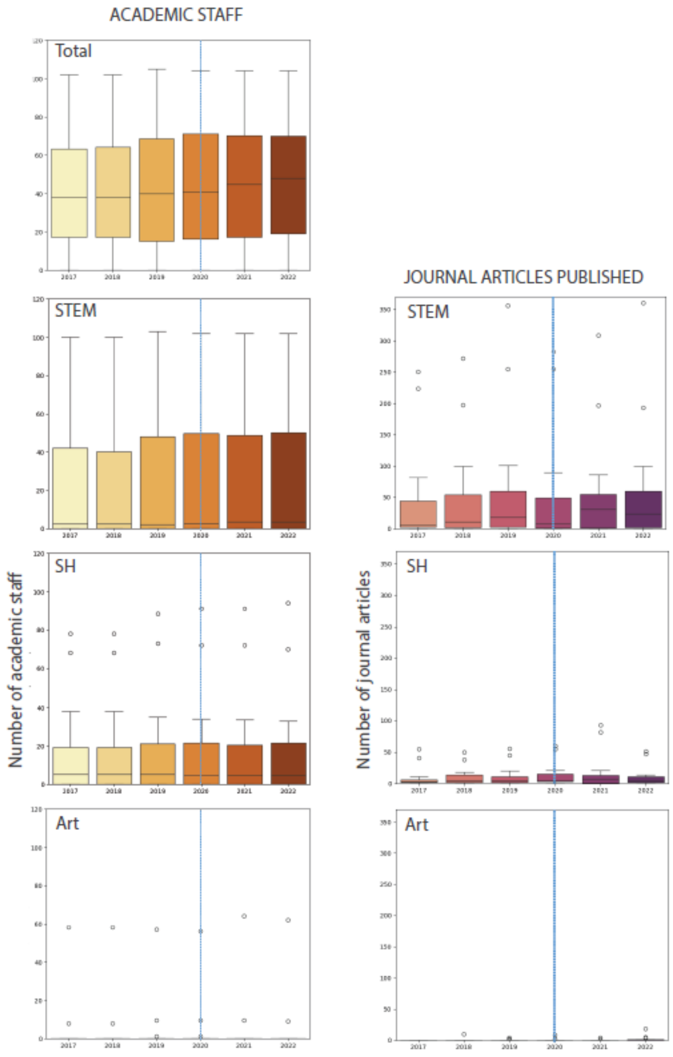

Figure S4. Financial indicators for the University of Split from 2017 to 2023 business year (in Euro). The indicators are calculated for calendar years. Blue vertical line indicates the start of the COVID-19 pandemic.

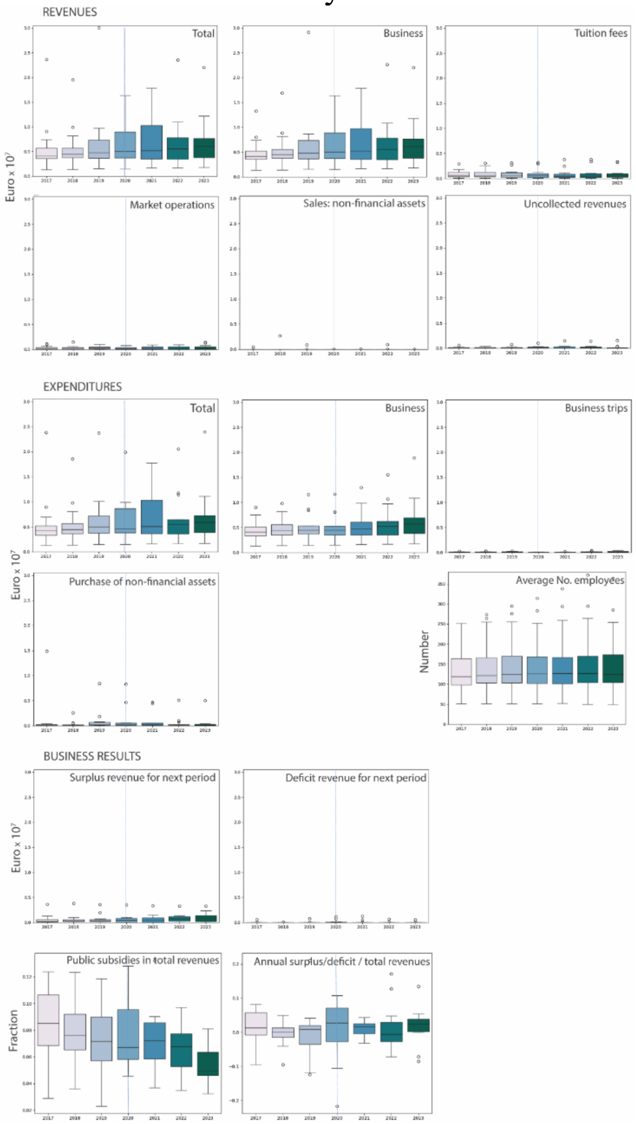

Figure S5. Outgoing and incoming staff and students mobilities from 2016/2017 to 2022/2023 academic year. Blue vertical line indicates the start of the COVID-19 pandemic.

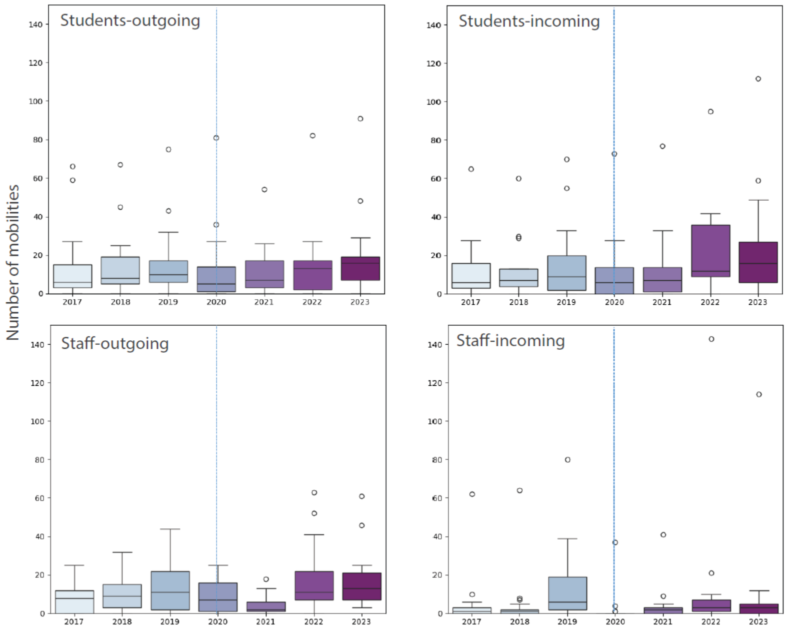

Supplement: Online Supplementary Document [file jogh-16-04017-s001.pdf]
